# Lipocalin-2-Mediated Insufficient Oligodendrocyte Progenitor Cell Remyelination for White Matter Injury After Subarachnoid Hemorrhage *via* SCL22A17 Receptor/Early Growth Response Protein 1 Signaling

**DOI:** 10.1007/s12264-022-00906-w

**Published:** 2022-07-07

**Authors:** Qiang Li, Xufang Ru, Yang Yang, Hengli Zhao, Jie Qu, Weixiang Chen, Pengyu Pan, Huaizhen Ruan, Chaojun Li, Yujie Chen, Hua Feng

**Affiliations:** 1 Department of Neurosurgery and State Key Laboratory of Trauma, Burn and Combined Injury, Southwest Hospital, Third Military Medical University (Army Medical University), Chongqing, 400038 China; 2 Department of Neurobiology, College of Basic Medical Sciences, Third Military Medical University (Army Medical University), Chongqing, 400038 China; 3 Model Animal Research Center, Nanjing University, Nanjing, 210032 China; 4 Chongqing Key Laboratory of Precision Neuromedicine and Neuroregenaration, Southwest Hospital, Third Military Medical University (Army Medical University), Chongqing, 400038 China; 5 Chongqing Clinical Research Center for Neurosurgery, Southwest Hospital, Third Military Medical University (Army Medical University), Chongqing, 400038 China

**Keywords:** White matter injury, Oligodendrocyte progenitor cell, Remyelination, Subarachnoid hemorrhage, Multiple sclerosis, Lipocalin-2, Early growth response protein 1

## Abstract

Insufficient remyelination due to impaired oligodendrocyte precursor cell (OPC) differentiation and maturation is strongly associated with irreversible white matter injury (WMI) and neurological deficits. We analyzed whole transcriptome expression to elucidate the potential role and underlying mechanism of action of lipocalin-2 (LCN2) in OPC differentiation and WMI and identified the receptor SCL22A17 and downstream transcription factor early growth response protein 1 (EGR1) as the key signals contributing to LCN2-mediated insufficient OPC remyelination. In LCN-knockdown and OPC EGR1 conditional-knockout mice, we discovered enhanced OPC differentiation in developing and injured white matter (WM); consistent with this, the specific inactivation of LCN2/SCl22A17/EGR1 signaling promoted remyelination and neurological recovery in both atypical, acute WMI due to subarachnoid hemorrhage and typical, chronic WMI due to multiple sclerosis. This potentially represents a novel strategy to enhance differentiation and remyelination in patients with white matter injury.

**Supplementary Information:**

The online version contains supplementary material available at 10.1007/s12264-022-00906-w.

## Introduction

White matter injury (WMI) causes deficits in movement, sensation, and cognition and occurs in individuals with typical demyelination disorders, such as multiple sclerosis (MS), amyotrophic lateral sclerosis, and experimental autoimmune encephalomyelitis [1]. Similarly, in patients with hemorrhagic stroke, including subarachnoid hemorrhage (SAH), WMI remains a pivotal factor that impedes functional recovery and results in an unsatisfactory prognosis after surgical rescue [2]. Recently, Gaastra *et al*. retrospectively analyzed data from the UK Biobank and found that cognitive dysfunction significantly impedes the return to work of patients with SAH [3]. Lee *et al*. found 44 abnormal WM tracts among 48 regions of interest in patients with SAH using functional magnetic resonance imaging [4]. Although WMIs have distinct etiologies, a shared neuropathological feature involves the failed differentiation of oligodendrocyte precursor cells (OPCs) into myelin-producing oligodendrocytes following injury, causing impaired myelin formation and regeneration (remyelination) [5]. Nevertheless, the mechanisms underlying this pathology have not been fully elucidated, as a comprehensive understanding of the inhibitory microenvironment that deters oligodendrocyte differentiation and regeneration is lacking; moreover, approved therapies are also lacking [6, 7].

In an SAH mouse model, Peng *et al*. found that oligodendrocyte death rapidly occurs in the corpus callosum (CC) and that the number of inherent OPCs transiently increased with endogenous repair, but OPC proliferation and remyelination are impaired one week after ictus [8]. Among the complex microenvironment in white matter (WM) after SAH, previous work has indicated that lipocalin-2 (LCN2) has a negative effect on remyelination in patients with progressive MS[9] and might represent a biomarker of acute brain injury [10, 11] and a “help-me” signal for repair [12]. In our previous study [13], we compared WM tissue between wild-type and LCN2-knockdown mice and found correlations between the expression of Cyr61, Olig1, Slc6a9, and other genes. Olig1 is a basic helix-loop-helix transcription factor involved in neurodevelopment and OPC remyelination [14, 15], as well as the only subtype of Olig protein involved in myelin repair [16, 17]. Moreover, we found remarkable proliferation and migration of OPCs after SAH, but remyelination was incomplete and insufficient to restore long-term neurological function, suggesting the presence of a potent inhibitory microenvironment that impedes oligodendrocyte differentiation and maturation following SAH and other conditions [18]. Hence, the questions of whether LCN2, as one component of the inhibitory microenvironment, inhibits remyelination by suppressing OPC differentiation, and how oligodendroglial lineage cell behavior is regulated remain to be fully elucidated.

In the present study, by comparing the results with MS as a positive control disease model, we reveal the roles of LCN2 in the remyelination and functional recovery of WMI after SAH. Furthermore, we identified early growth response protein 1 (EGR1) as the pivotal downstream factor of LCN2/SCL22A17 receptor-mediated insufficient OPC remyelination, revealing a novel strategy for potential clinical intervention in myelin disorders throughout the lifespan.

## Materials and Methods

### Animals

All animal protocols were approved by the Laboratory Animal Welfare and Ethics Committee of the Third Military Medical University (Approval No. AMUWEC2020793). All animal studies were conducted in accordance with the China Public Health Service Policy on the Humane Care and Use of Laboratory Animals. Mouse colonies were maintained in accordance with institutional guidelines. C57BL/6 mouse colonies were established from mice initially purchased from the Animal Center of the Third Military Medical University (Permit No. SCXK20170002; Chongqing, China). PDGFRα-Cre mice were purchased from the Jackson Laboratory (Cat log: 018280, Bar Harbor, ME). EGR1^fl/fl^ mice were kindly provided by the Model Animal Research Center of Nanjing University. LCN2^KD^ mice were generated by injecting an LCN2 siRNA, which induced stable knockdown in C57BL/6 mice. The mice were housed in an environment with a 12-h light/dark cycle and allowed free access to food and water throughout the experimental period. The power was calculated with a two-sided 95% confidence interval *via* the normal approximation method using SPSS 13.0 software to limit the use of animals and >80% power (84%–100%) was achieved for all experiments. ARRIVE guidelines were followed when providing the details of the experiments and quantifications and in reporting.

### siRNA Administration

Using previously-described methods [19, 20], siRNA was administered by intracerebroventricular injection. Briefly, anesthetized mice were placed on a stereotaxic apparatus (RWD Life Science, Shenzhen, China), and the bregma was exposed. A burr hole was drilled into the bone over the left hemisphere, at the coordinates 1.5 mm lateral, 3.4 mm posterior, and 3.5 mm below the horizontal plane of the bregma. A 2-μL volume of different siRNAs was delivered into the amygdala with a Hamilton syringe (Hamilton Co., Reno, NV). The siRNAs were: 1 μg/μL LCN2 siRNA, EGR1 siRNA, 24q3r (SLC22A17) siRNA, or the respective scrambled siRNA (scr siRNA) (Santa Cruz Biotechnology, Shanghai, China). Two injections were made at 12 and 24 h before SAH to enhance the silencing effect. The sequences are provided in the Supplemental Material (Reagents List).

### Mouse SAH

The endovascular perforation model of SAH was established in mice as reported previously [18, 21, 22]. Briefly, mice were anesthetized with a 3% isoflurane/air mixture in a rodent respirator (Harvard Apparatus, Holliston, MA). A sharpened 5-0 monofilament nylon suture was inserted rostrally into the left internal carotid artery from the external carotid arterial stump and perforated the bifurcation of the anterior and middle cerebral arteries. Sham-operated mice underwent the same procedures except that the suture was withdrawn without puncture. All animals were housed at 22 °C–25°C and 65%–70% humidity on a 12-h light/dark cycle and were provided sufficient food and water. Blood pressure and heart rate were non-invasively monitored during the operation *via* the tail. The severity of SAH was blindly assessed in all animals after they were sacrificed, as previously described [23]. Each animal received a total score by summing the scores. Only SAH mice with moderate hemorrhage (scores of 8–12) were included in the experiments.

### Cuprizone Diet-Induced MS

Experimental demyelination was induced by feeding male LCN2^*KD*^ mice and their wild-type littermates a diet containing 0.2% cuprizone (biscyclohexanone oxaldihydrazone, Sigma–Aldrich, Shanghai, China) mixed into standard rodent chow [24]. Cuprizone feeding started at postnatal day 56 (P56) and was maintained for 5 weeks to induce profound demyelination. Spontaneous remyelination was enabled by withdrawing cuprizone from the diet. During experimentally-induced demyelination and remyelination, the mice were observed daily, and their body weight was measured twice per week.

### Magnetic Resonance Imaging

Magnetic resonance imaging (MRI) was applied using a 7.0 T small animal magnetic resonance system (PharmaScan; Bruker, Ettlingen, Germany). Mice were anesthetized with 1% isoflurane (Shandong Keyuan Pharmaceutical Co. Ltd., Shandong, China), and the heart rate was maintained at ~100 bpm. At 7 days after SAH modeling or cuprizone withdrawal, T2-weighted turbo spin-echo sequences (T2WI) were collected with the following parameters: slice thickness, 1 mm; slices, 15; inter-slice distance, 1 mm; repetition time, 3 s; averages, 1; matrix size, 256 × 256; flip angle, 180°; and total scan time for image acquisition*,* 1 min 20 s. Five reference images were obtained at 14 days with the following parameters: field of view, 2 cm; slice thickness, 0.6 mm; slices, 20; matrix size, 128 × 128; repetition time, 5 s; and averages, 2. The WMI volume was calculated with ImageJ software (National Institutes of Health, Bethesda, MD) using the following equation: injury size % = 100 × Σ {injury area − (ipsilateral hemisphere − contralateral hemisphere)}/Σ (contralateral hemisphere). Fractional anisotropy was measured from the tensor map in the ipsilateral internal capsule with ParaVision 5.0 software (Bruker, Ettlingen, Germany).

### Evaluation of Neurological Function

*mNSS:* As previously reported [25], neurological functions were evaluated using the modified neurological severity score (mNSS) at 1, 3, 5, 7, 9, 12, and 14 days after SAH. The mean of the neurological scores determined by two blinded observers was calculated.

*BMS:* The recovery of hindlimb motor function was scored using the Basso mouse scale (BMS) open-field locomotor rating scale, which was developed specifically for mice [26]. The score ranged from 0 (complete paralysis) to 9 (normal mobility), and the number of errors at each footstep was measured for 100 steps. Mice were observed individually for 2 min in an open field.

*Morris Water Maze:* The Morris water maze was applied to evaluate hippocampus-dependent spatial learning and memory in mice [27]. A large circular tank (0.8 m diameter, 0.4 m depth) was filled with water (25 °C ± 1°C, 20 cm depth), and the escape platform (8×4 cm) was submerged 1 cm below the surface. Each section was monitored using a video capture system. The escape latency and trajectory of swimming were recorded for each mouse. The hidden platform was located at the center of one of the four quadrants in the tank. The location of the platform was fixed throughout testing. Mice had to navigate using extra-maze cues that were placed on the walls of the maze. From days 1 to 4, mice underwent three trials with an inter-trial interval of 5 min. The mice were randomly placed in the tank facing the sidewall at one of the four start locations and allowed to swim until they found the platform or for a maximum of 120 s. Each mouse that failed to find the platform within 120 s was guided to the platform. The animal then remained on the platform for 20 s before being removed from the pool. The day after the hidden platform training, a probe trial was conducted to determine whether mice used a spatial strategy to find the platform. On day 5, the platform was removed from the pool, and the mouse was allowed to swim freely for 120 s. The time spent (escape latency), swimming distance, and the number of times the mouse crossed the former position of the hidden platform in each quadrant of the pool were recorded.

### Black Gold II Myelin Staining

The Black Gold II staining kit (Merck Millipore, Chengdu, China) was used according to the manufacturer’s instructions [28]. Briefly, 50-μm paraformaldehyde (PFA)-fixed brain sections were mounted onto Superfrost Plus slides (Fisher Scientific, Shanghai, China). Coronal brain sections were initially air dried and then rehydrated and transferred to a lukewarm 0.3% Black Gold II solution. After color development (10 min), the slides were rinsed in a 1% sodium thiosulfate solution at 60°C, dehydrated, and mounted with Permount. The integrated staining intensity in several brain areas was assessed using ImageJ. Data represent the pooled results from at least four brains per experimental group. Ten slices per brain were used, and quantification was performed by researchers who were blinded to the genotype of the sample.

### Transmission Electron Microscopy Analysis

Mice were intracardially perfused with 4% (w/v) PFA and 2% (v/v) glutaraldehyde (Aladdin Industrial Corp., Shanghai, China) in 0.1 mol/L phosphate buffer. Tissue was postfixed overnight at 4°C and transferred to 1% (v/v) glutaraldehyde until embedding. Tissue sections (1 mm were processed into araldite resin blocks. In addition, 1-μm sections cut on a microtome were stained with a 1% toluidine blue/2% sodium borate solution prior to bright-field imaging at 100× magnification using a Zeiss AX10 microscope. The number of myelinated axons was counted in 50 μm × 50 μm images of the corpus callosum in a blinded manner, in 2–4 sections per mouse, and then the values were averaged. Ultrathin sections (60 nm) were cut from the corpus striatum and stained in uranyl acetate and lead citrate, and the grids were imaged using a JEOL transmission electron microscope. Axon diameter, myelin thickness, and axonal thickness was calculated from the measured area based on the assumption of circularity using ImageJ (diameter = 2 ×$\sqrt{{\text{area}}/\uppi }$), and a minimum of 100 axons per animal were analyzed. G-ratios (the ratio of axon diameter to axon plus myelin sheath diameter) were calculated using ImageJ for at least 100 axons per animal. The axonal thickness was calculated by subtracting the axon diameter from the diameter of the innermost compact layer.

### Enzyme-linked Immunosorbent Assay (ELISA) of LCN2

At 3 days after SAH, mouse brains were separated into WM, cortex (CTX), and CC. These mouse tissues were homogenized in phosphate-buffered saline supplemented with 1 mmol/L phenyl-methylsulfonyl fluoride and 1 mmol/L ethylene glycol tetra-acetic acid. Samples were diluted 1:2 in sample diluent and then incubated on coated microtiter plates (96-well flat bottom) for 24 h. Levels of LCN2 were determined using commercially available ELISA kits (Jonln Bio, Shanghai, China).

### Focal White Matter Injury (FWMI)

Focal WM demyelinating lesions were induced in the striatum of 12-week-old male C57BL/6 mice by administering a stereotaxic injection of 4 μL of 0.01% (v/v) ethidium bromide (Sigma–Aldrich, Shanghai, China) using a Hamilton syringe. At 5 and 10 days after injection (dpi), mice were intracardially perfused with 4% PFA and brains were cryoprotected, cryosectioned, and stained as above. FWMI in non-lesioned tissue served as a control.

### OPC Cultures

Mixed glial cultures of OPCs were generated from both female and male C57BL/6 mouse pups at P0–P2, as previously reported[29]. The mixed cultures were placed on a rotary shaker at 37°C at 250 rpm for 1 h to allow microglia to adhere. OPCs were subsequently isolated from the floating fraction after 16 h of incubation on a rotary shaker, and the contaminating astrocytes were depleted by differential adhesion. OPCs were plated in DMEM/F12 containing 50 μg/mL apo-transferrin, 30 nmol/L sodium selenite, 0.1% (w/v) bovine serum albumin (BSA), 60 ng/mL progesterone, 16 μg/mL putrescine, 10 μg/mL insulin, 100 μg/mL BSA fraction V, 400 ng/mL L-thyroxine, 4.5 g/L glucose, 1% (v/v) L-glutamine, 1% (v/v) pyruvate, 1% penicillin/streptomycin, 10 ng/mL platelet-derived growth factor, and 10 ng/mL fibroblast growth factor-2 (all from Sigma–Aldrich, Shanghai, China). OPCs were plated at a density of 2 × 10^4^ cells per well in 6-well polylysine (0.1 μg/mL)-coated plates (Corning Inc., Corning, NY) or confocal plates (glass bottom dish, Cellvis, Mountain View, CA). OPCs were treated with LCN2 (1, 2.5, 5, 10, 20, 40, 80, or 160 ng/mL, R&D Systems, Shanghai, China) or vehicle control (0.0002% BSA) for 1 day. In a subset of experiments, OPCs were treated with 400 ng/mL triiodothyroxine (T3) and LCN2 (40 ng/mL) or vehicle or plus 5 ng/mL EGR1 siRNA and scr siRNA control. In the experiments designed to determine the molecular mechanism, OPCs were treated with 5% (v/v) fetal bovine serum (FBS), 10 μmol/L oxyhemoglobin (OxyHb), 50 nmol/L FeCl_2_, 5 ng/mL 24q3r (SLC22A17) siRNA, and scr siRNA control and their respective combinations. Cells were matured to oligodendrocytes by withdrawing growth factors from the medium. For CNPase intensity measurements, O4 and CNPase staining were measured in square pixels (px^2^) with a threshold of 2000 px^2^ set to exclude background/false-positives. An average of 100 cells were counted per image, with 2 images assessed per condition per biological replicate (>600 cells were quantified per condition); values were averaged for each biological sample.

### Microarray Analysis

Human OPCs (ScienCell Research Laboratories, Carlsbad, CA) were grown in oligodendrocyte precursor cell medium (ScienCell Research Laboratories, Carlsbad, CA), treated with LCN2 or vehicle for 24 h, and harvested. Total RNA was extracted with TRIzol (Invitrogen, Shanghai, China). RNA was further purified, and genomic DNA was eliminated using the RNEasy Plus mini kit (Qiagen, Shanghai, China). Human GeneChip® Whole Transcript Expression microarrays were from Affymetrix®. Four independent biological replicates were assayed per group. Sample labeling and array hybridization were conducted by Shanghai Qimin Biotech according to Affymetrix procedures. Data were analyzed with Partek GS using the Affymetrix extended probeset annotation with a GC background-correction RMA algorithm. Differentially-expressed mRNAs and probesets were identified using a two-sample* t* test, with an FDR of 0.1, unless indicated otherwise.

### Qualitative Reverse Transcription PCR (qPCR)

Total RNA was extracted from cultured OPCs or brain tissue using the technique described previously by our group [30]. Afterward, cDNAs were synthesized using the High-Capacity cDNA Archive Kit (Qiagen, Shanghai, China). For qPCR, 2 ng of cDNAs was used, and the CFX96 Touch Real-Time PCR Detection System (Bio–Rad, Shanghai, China) was used for analysis under the following conditions: 95°C for 20 s, 95°C for 1 s, and 60°C for 20 s for 45 cycles (values >40 cycles were defined as not expressed). All genes were analyzed in triplicate, and each run contained the reference gene (GAPDH) as an internal control to normalize the expression of the target genes. The primers used in the qPCR assay are listed in the Major Resources Tables. The ΔCT of 'normal' controls was subtracted from the ΔCT of the LCN2-treated group to determine the differences (ΔΔCT) and fold change (2^-ΔΔCT) in gene expression. Gene expression was illustrated as the log10-fold change value compared to control.

### Genotyping

Genomic DNA was extracted from tail tissue using the Wizard SV genomic purification system (Promega Biotech, Beijing, China) according to the manufacturer’s instructions. EGR1 floxed mice were genotyped using PCR strategies described previously [31]. Briefly, EGR1 floxed mice were genotyped using the primers P1 (CCT TTC CTC ACT CAC CCA CCA TGG) and P2 (CAC CCA CGC AGC TTG AGT TCT C). PDGFRa-Cre mice were genotyped using the primers F (TCA GCC TTA AGC TGG GAC AT) and R (ATG TTT AGC TGG CCC AAA TG). Cre-mediated recombination was detected using P4 (CAA ATG TTG CTT GTC TGG TG) and P5 (GTC AGT CGA GTG CAC AGT TT).

### Analysis of OPC Proliferation with CCK-8

According to the manufacturer’s instructions, CCK-8 (Beyotime Biotechnology, Shanghai, China) was used to measure the proliferation of OPCs [32]. First, 1×10^3^ OPCs were plated in a volume of 100 µL into each well of two 96-well plates. After incubation with 0 ng/µL, 1 ng/µL, 2.5 ng/µL, 5 ng/µL, 10 ng/µL, 20 ng/µL, 40 ng/µL, 80 ng/µL, or 160 ng/µL LCN2 for 24 h, the cells were incubated with the yellow CCK-8 solution (10 µL) for ~2 h. After this incubation period, an orange soluble formazan product formed. The formazan product was spectrophotometrically quantified using an enzyme-linked immunosorbent assay reader (λ = 450 nm), and growth curves were constructed. All assays were performed in quadruplicate

### Wound Healing Assay of OPC Migration

As previously reported [33], OPCs were cultured in 24-well plates (Corning Inc., Corning, NY) and co-incubated with 0 ng/µL, 1 ng/µL, 2.5 ng/µL, 5 ng/µL, 10 ng/µL, 20 ng/µL, 40 ng/µL, 80 ng/µL, or 160 ng/µL LCN2 for 24 h. After the cells reached semi-confluence, a wound was created in the cell monolayer using a yellow pipette tip. OPCs were cultured in DMEM supplemented with 2% FBS for 24 h with or without various concentrations of LCN2. Scratched fields were imaged every 2 h and were monitored with a Zeiss Live-Cell Imaging System (Carl Zeiss, Jena, Germany) and Axion Version software (Axion Biosystems, Atlanta, GA).

### Fluorescent Immunohistochemistry

Brain tissue or spinal cord sections (10 μm thick) and cultured OPCs were stained using fluorescent immunohistochemistry. Sections or cells on confocal plates were fixed with 4% PFA, and membrane proteins were solubilized with 0.3% Triton X-100 for 30 min. Antigen retrieval, if necessary, was performed by treatment with 95% formic acid for 5 min, followed by boiling in citrate buffer (10 mmol/L sodium citrate and 0.05% Tween-20; pH 6). Sections or confocal plates were blocked with 10% normal goat serum and incubated overnight at 4°C with a combination of antibodies. All primary antibodies were diluted in 4% normal goat serum in PBS. Sections or confocal plates were then incubated for 2 h at room temperature with appropriate fluorescent dye-conjugated secondary antibodies. Sections or cells on confocal plates were counterstained with DAPI (Lineage Cell Therapeutics, Alameda, CA) and coverslipped (PermaFluor, Thermo Fisher Scientific, Shanghai, China). The intensity of myelin basic protein (MBP) expression was measured by analyzing MBP staining in px^2^, with a threshold of 2000 px^2^ set to exclude background/false-positives. The antibodies are provided in the Supplemental Material (Reagents List).

### Western Blotting

Cultured OPCs or brain samples were lysed with RIPA buffer (Thermo Fisher Scientific, Shanghai, China) supplemented with 1% protease inhibitor cocktail set III ethylenediaminetetraacetic acid-free (Roche, Shanghai, China). Protein concentrations were determined using a Pierce BCA Protein Assay Kit (Lineage Cell Therapeutics, Alameda, CA) according to the manufacturer’s instructions. Samples were diluted in loading buffer (Lineage Cell Therapeutics, Alameda, CA) containing 5% β-mercaptoethanol (Aladdin Industrial Corp,, Shanghai, China) and heated at 100°C for 5 min; 10 μg of protein were loaded onto an acrylamide gel (6%–15%; Bio–Rad). Gel electrophoresis was performed in Tris-glycine-sodium dodecyl sulfate running buffer (Lineage Cell Therapeutics, Alameda, CA) at 100 V, and proteins were transferred to polyvinylidene difluoride membranes (Merck Millipore, Chengdu, China) for 2 h at 150 mA in 10% transfer buffer [3% Tris–HCl (Aladdin Industrial Corporation, Shanghai, China), 15% glycine (Solarbio Life Sciences, Beijing, China), pH 8.3, and 20% methanol (Shanghai Songon, Shanghai, China) diluted in water]. Membranes were blocked with 5% BSA in Tris-buffered saline (TBST) [4% NaCl, 0.1% KCl, 1.5% Tris–HCl, and 0.1% Tween-20 (all from Aladdin Industrial Corp., Shanghai, China), pH 7.4] for 1 h at room temperature on a horizontal shaker and then incubated overnight at 4°C with primary antibodies. The membranes were washed three times with TBST for 5 min and incubated with horseradish peroxidase (HRP)-IgG secondary antibody conjugates (1:2000; ZSGB-Bio, Beijing, China) for 1 h at room temperature. A chemiluminescent substrate detection reagent, WesternBright Quantum HRP substrate (Advansta, Menlo Park, CA), was used to visualize bands. For a loading control, all membranes were re-blotted with anti-mouse or anti-rabbit GAPDH antibodies. The antibodies are described in the Supplemental Material (Reagents List).

### Statistical Analyses

Data handling and statistical processing were performed using Microsoft Excel and GraphPad Prism 6.0 software. Data are presented as the mean ± SEM. Power for sample size was calculated using OpenEpi, and a power ranging from 84% to 100% was obtained for all experiments. All cell counts and analyses were performed by researchers who were blinded to the experimental treatment. Statistical tests included a one-sample* t* test for data where the values were normalized to the control and a two-tailed Student’s *t* test when only two groups were compared. The multiple *t* test was performed when comparing data between 2 independent groups. Nonparametric one-way ANOVA with Dunn’s multiple comparisons *post hoc* test was applied when >3 comparisons were made, and one-way ANOVA with Bonferroni's multiple comparisons *post hoc* test was applied when ≤3 comparisons were made. Two-way ANOVA with Sidak's multiple comparisons test was used for inter-group comparisons between grouped data. The slopes of myelin thickness *versus* axon diameter were compared using the Extra Sum of Squares F test. Curve distributions of the proportion of myelinated axons per axon diameter were compared using the Kolmogorov–Smirnov test. *P <*0.05 was considered statistically significant.

## Results

### Enrichment of LCN2 Impedes Functional Recovery after WMI

**Fig. 1:**
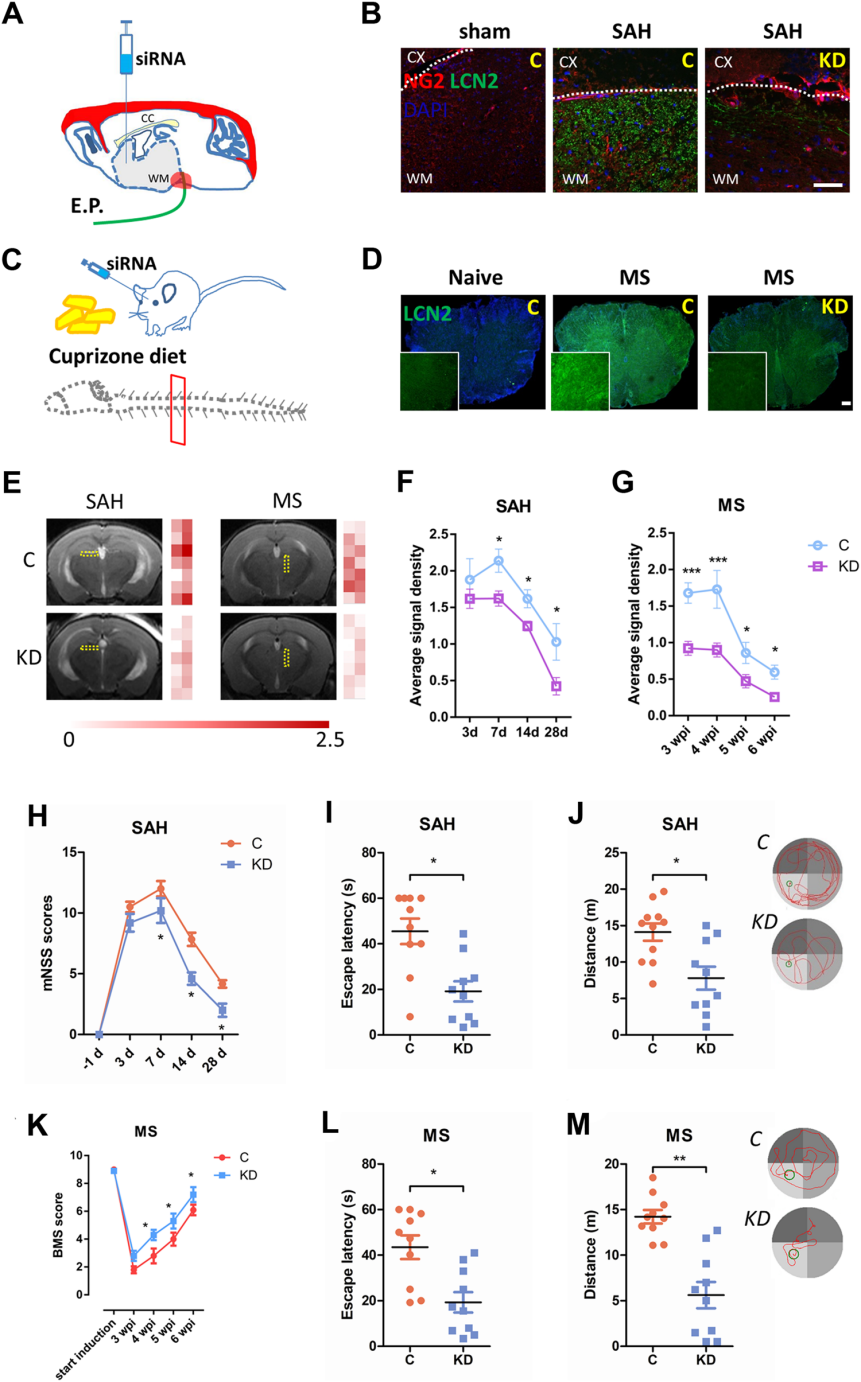
Enrichment of LCN2 impedes functional recovery after WMI. **A** Schematic of the endovascular perforation-induced SAH model and injection of siRNA. **B** Images of NG2 (red) and LCN2 (green) expression in the brains of control and LCN2^*KD*^ mice at 7 days after sham or SAH surgery (scale bar, 50 μm). **C** Schematic of the cuprizone diet-induced MS model and injection of siRNA. **D** Images of LCN2 (green) expression in the spinal cords of control and LCN2^*KD*^ mice at 3 wpi of MS (scale bar, 50 μm). **E** MRI (T2WI) of control and LCN2^*KD*^ mice at 7 days after SAH and at 3 wpi of MS. The colored grid bar at the right of each panel shows the T2WI signal in the yellow frame, ranging from 0 to 2.5. **F** Average signal density of the white matter in control and LCN2^*KD*^ mice (*n =* 3 mice per time point) at 3, 7, 14, and 28 days after SAH (*P <0.05 two-way ANOVA with Sidak’s multiple comparisons test). **G** Average signal density of the spinal cord white matter in control and LCN2^*KD*^ mice (*n =* 3 per time point) at 3, 4, 5, and 6 wpi of MS (*P <0.05, ***P <0.001, two-way ANOVA with Sidak's multiple comparisons test). **H** mNSS scores for control and LCN2^*KD*^ mice (*n =* 6 per time point) at –1 (1 day before SAH induction), 3, 7, 14, and 28 days after SAH (*P <0.05, two-way ANOVA with Sidak's multiple comparisons test). **I** Escape latency before locating the platform of control and LCN2^*KD*^ mice (*n =* 10 mice per group) with SAH (*P = 0.0342, two-tailed Student’s *t* test). **J** Swimming distance before locating the platform of control and LCN2^*KD*^ mice (*n =* 10 mice per group) with SAH (*P = 0.0406, two-tailed Student’s* t* test). **K** BMS scores of control and LCN2^*KD*^ mice (*n =* 6 mice per time point) at 0 (induction), 3, 4, 5, and 6 wpi of MS (*P <0.05, two-way ANOVA with Sidak's multiple comparisons test). **L** Escape latency before locating the platform of control and LCN2^*KD*^ mice (*n =* 10 mice per group) with SAH (*P = 0.0212, two-tailed Student’s *t* test). **M** Swimming distance before locating the platform of control and LCN2^*KD*^ mice (*n =* 10 mice per group) with SAH (**P = 0.0052, two-tailed Student’s* t* test).

Using typical WM disorder (MS) and atypical WM disorder (SAH) models, we sought to evaluate whether LCN2 is a shared pathological molecule that is overexpressed after WMI. First, an endovascular perforation was established to induce SAH, and a cuprizone diet was used to induce an MS model (Fig. 1A, C). According to immunofluorescence staining, LCN2 expression was increased in the brains (especially in the WM area) of SAH mice and in the spinal cords of MS mice (Fig. 1B, D). Upregulation of LCN2 was confirmed not only in the acute phase of SAH (within 2 days) but also over a longer period following SAH (up to 14 days), as determined using western blot analysis (Fig. S1E).

Next, we established the effective knockdown of LCN2 through siRNA injection to further assess whether blocking this increase in LCN2 expression promoted the recovery of WM function (Fig. 1A, C). LCN2 knockdown significantly inhibited its increased expression in the WM (Fig. 1B, D) and reduced LCN2 protein levels in the cortex and CC, as determined using western blotting and ELISA (Fig. S1F, G).

To verify if inhibition of LCN2 reduced WMI, we detected the T2 signal in the brains of mice from the MS and SAH groups using 7.0 T magnetic resonance imaging (Fig. 1E). The average T2 signal density, which predicts brain injury, was significantly reduced in the CC of SAH mice and in the WM of MS mice after LCN2 knockdown (Fig. 1F). Moreover, the WMI volume decreased in LCN2^*KD*^ mice after SAH (Fig. S1C). Nonetheless, LCN2 knockdown did not affect re-bleeding or hematoma absorption because the hemorrhagic severity of endovascular perforation-induced SAH was not significantly altered, as evidenced by the bleeding volume score and MRI (Fig. S1A, B). The secretion of LCN2 increased as the hemorrhagic score increased in the WM at 3 days after SAH (Fig. S1D).

Finally, to determine whether LCN2 inhibition promoted functional recovery after WMI, SAH and MS mice were performed with locomotor and cognitive test. LCN2 knockdown reduced the mNSS scores of SAH mice at 7, 14, and 28 days and improved BMS scores at 4, 5, and 6 weeks after MS (Fig. 1G, J). Furthermore, LCN2 knockdown significantly shortened the escape latency and distance traveled before locating the underwater platform (Fig. 1H, I, K, L).

Overall, these data suggested that the upregulation of LCN2 impedes functional recovery after WMI in both SAH and MS mice.

### Inhibition of LCN2 Promotes Remyelination and Myelin Maturation After WMI

Having shown the positive effects of LCN2 knockdown on functional recovery after WMI, we next asked whether the inhibition of LCN2 promoted remyelination.

**Fig. 2:**
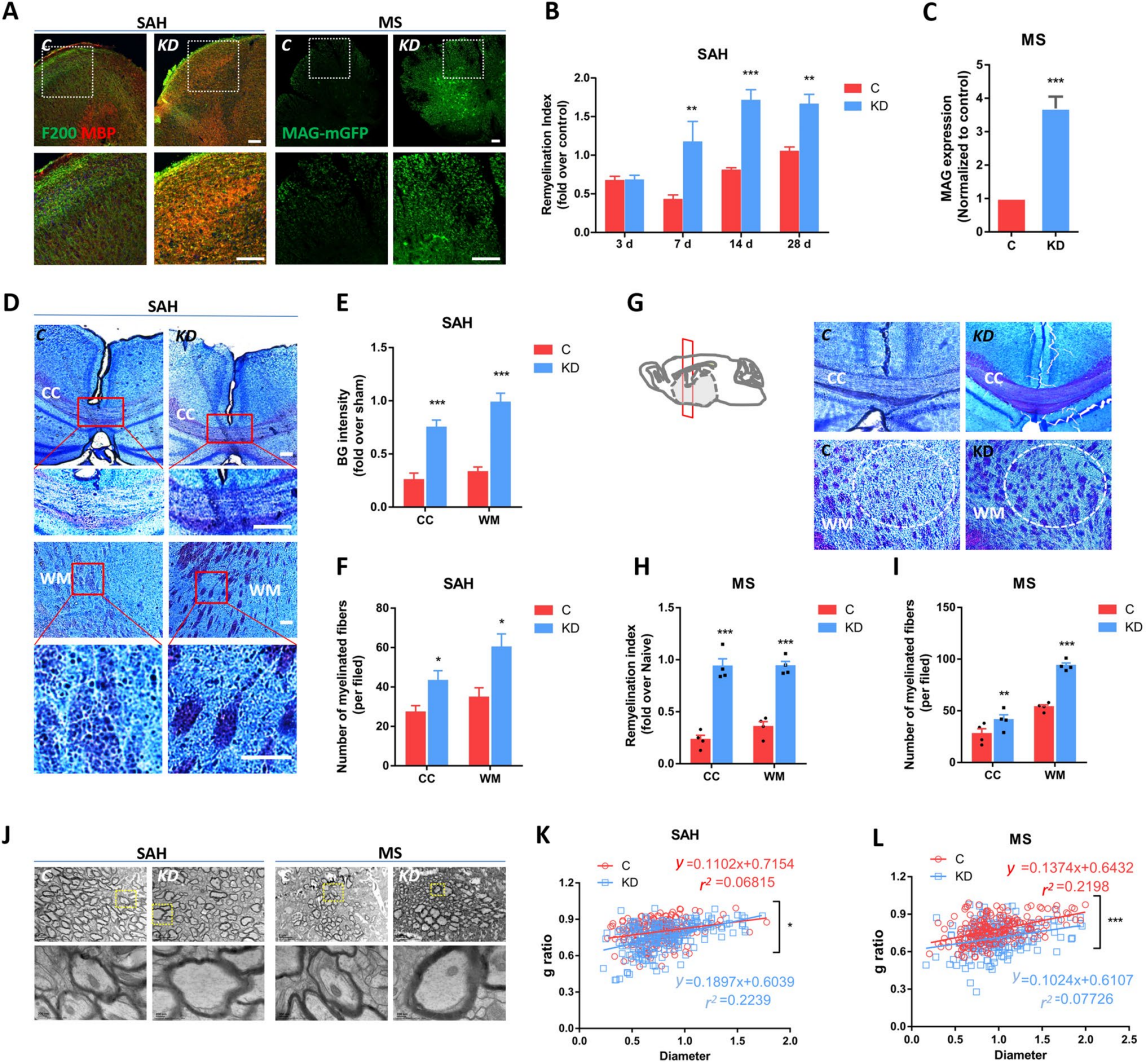
Inhibition of LCN2 promotes remyelination and myelin maturation after WMI. **A** Images of NF200 and MBP staining in the white matter (WM) of control and LCN2^*KD*^ mice at 7 days after SAH, and an expanded view of the white frame. Images of MAG expression (green) in the spinal cord WM of control and LCN2^*KD*^ mice at 5 wpi of MS, and an expanded view of the white frame (scale bars, 50 μm). **B** Remyelination index (co-localization of MBP and NF200, normalized to the area of NF200) of control and LCN2^*KD*^ mice (*n =* 5 fields per time point) at 3, 7, 14, and 28 days after SAH (**P <0.01, ***P <0.001 *vs* control group, one-way ANOVA with Dunnett's multiple comparisons test). **C** Normalized MAG expression in control and LCN2^*KD*^ mice at 5 wpi of MS (*n =* 5 fields per time point) (***P = 0.0003, two-tailed Student’s *t* test). **D** Black Gold (BG) II staining of the brains from control and LCN2^*KD*^ mice at 7 days after SAH, and expanded views of the corpus callosum (CC) and WM (scale bars, 75 μm). **E** Relative BG intensity in the CC and WM of control and LCN2^*KD*^ mice (*n =* 5 fields per time point) at 7 days after SAH (***P <0.001 *vs* control group, one-way ANOVA with Dunnett's multiple comparisons test). **F** Number of myelinated fibers in the CC and WM of control and LCN2^*KD*^ mice (*n =* 5 fields per time point) at 7 days after SAH (*P <0.05 *vs* control group, one-way ANOVA with Dunnett's multiple comparisons test). **G** BG II staining of the CC and WM of control and LCN2^*KD*^ mice at 5 wpi of MS (scale bar, 75 μm). **H** Normalized remyelination index in the CC and WM of control and LCN2^*KD*^ mice (*n =* 5 fields per time point) at 5 wpi of MS (***P <0.001 *vs* control group, one-way ANOVA with Dunnett's multiple comparisons test). **I** Number of myelinated fibers in the CC and WM of control and LCN2^*KD*^ mice (*n =* 5 fields per time point) at 5 wpi of MS (**P <0.01, ***P <0.001 *vs* control group, one-way ANOVA with Dunnett's multiple comparisons test). **J** Ultrastructure of the WM of control and LCN2^*KD*^ mice at 7 days after SAH and at 5 wpi of MS. The areas bounded yellow frames are magnified below (scale bars, 2 μm or 200 nm). **K** G-ratio *versus* axon diameter in control and LCN2^*KD*^ mice at 7 days after SAH (* *P =* 0.00323, extra sum of squares F test between slopes). **L** G-ratio *versus* axon diameter in control and LCN2^*KD*^ mice at 5 wpi at MS (***P = 0.0001, extra sum of squares F test between slopes).

In the present study, the inhibition of LCN2 reduced the expression of degraded MBP, a biomarker of demyelination, while regulating MBP expression in the WM of LCN2^*KD*^ mice after SAH (Fig. S2A, B). Nevertheless, the role of LCN2 in myelination is controversial because some studies indicate that it is a neuroprotective factor [34]. Surprisingly, the double-labeling results showed that LCN2 knockdown significantly increased remyelination at 7, 14, and 28 days post-SAH compared with scrambled control treatment, as measured by the remyelination index (co-localization of MBP and axonal neurofilament 200 (NF200) divided by the area of neurofilaments) (Fig. 2A, B). Black Gold II staining provided morphological confirmation, as LCN2^*KD*^ mice showed more myelinated fibers and a higher Black Gold intensity than control mice (Fig. 2D–F). Similarly, in the MS model, an increase in the level of MAG, an oligodendrocyte maturation-associated gene, was found in LCN2^*KD*^ mice compared to control mice (Fig. 2A, C).

As LCN2 was considered a promising prognostic biomarker for patients with early-stage MS [35], we next used Black Gold II staining to determine whether the knockdown of LCN2 also promoted remyelination after MS. The results showed an increased number of myelinated fibers and therefore an increase in the remyelination index in the CC and WM at 5 weeks after MS (Fig. 2G–I). We analyzed the ultrastructure of axons and myelin integrity in the WM to determine whether LCN2 knockdown affected the myelin sheath during the remyelination process. Our previous study revealed that SAH decreases the myelin thickness in the CC and WM during the early phase [18]. At 7 days after SAH in LCN2^*KD*^ mice, we measured increased myelin thickness and a statistically significantly reduced mean g-ratio, a measure of myelin sheath thickness calculated as the ratio of axon diameter to myelinated fiber diameter, in LCN2^*KD*^ mice compared to scrambled control-treated mice (Fig. 2J, K). At 5 weeks after MS, the mean g-ratio of the myelinated axons in LCN2^*KD*^ mice was significantly lower than that of control mice, indicating that the myelin sheaths were thicker in LCN2^*KD*^ mice (Fig. 2J, L).

**Fig. 3:**
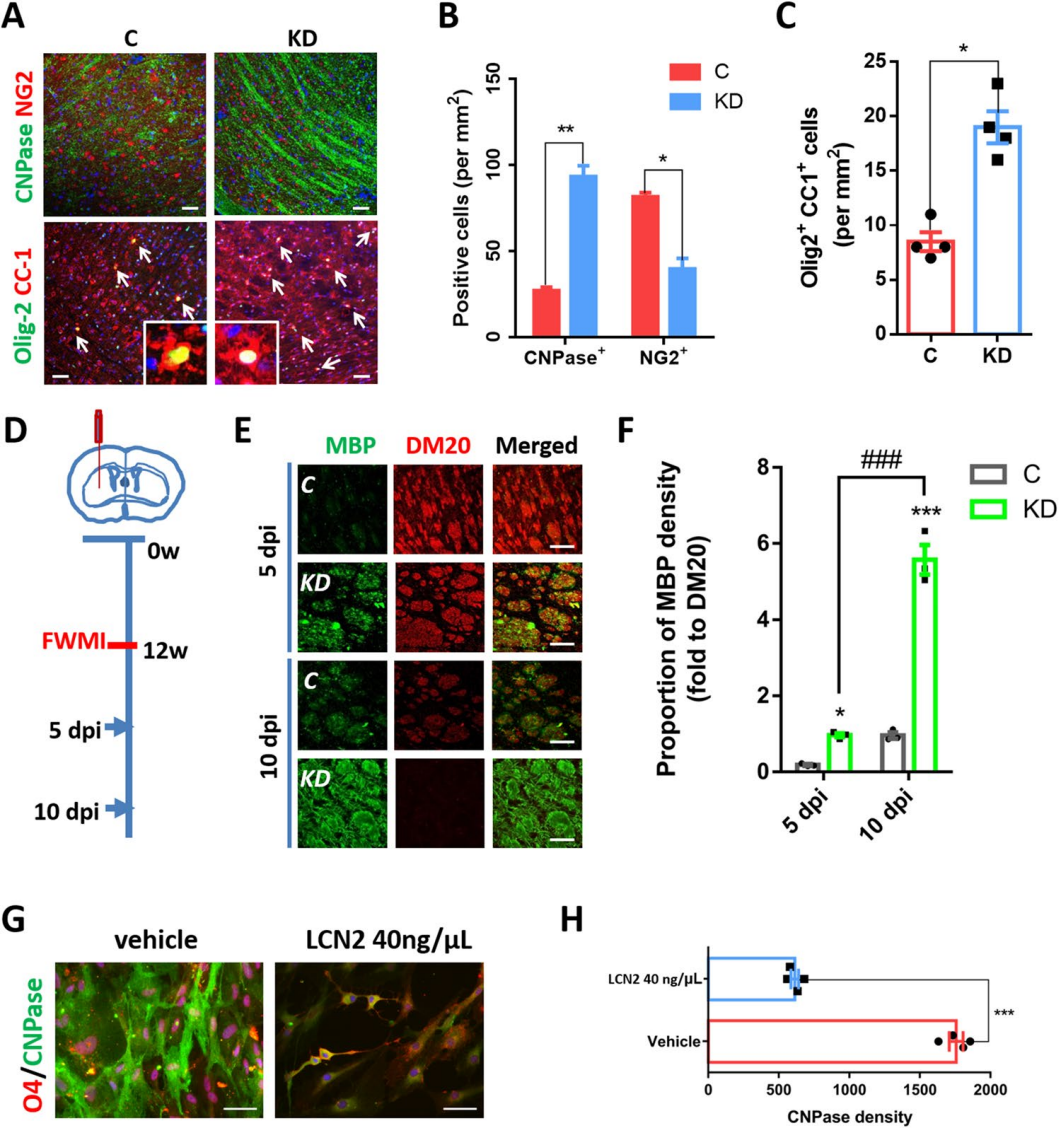
LCN2 inhibits OPC differentiation. **A** Numbers of OPCs (NG2+), oligodendrocytes (CNPase+), and mature oligodendrocytes (CC1+Olig2+) in the WM of control and LCN2^*KD*^ mice at 7 days after SAH. Inset, arrows indicate CC1+Olig2+ cells (scale bars, 50 μm). **B** Cells positive for CNPase and NG2 in control and LCN2^*KD*^ mice at 7 days after SAH (*n =* 4 fields per group). ***P = 0.0005 (CNPase+), **P = 0.0031 (NG2+), multiple* t* test. **C** Numbers of mature oligodendrocyte lineage cells (CC1+Olig2+) in control and LCN2^*KD*^ mice at 7 days after SAH (*n =* 4 fields per group). ***P = 0.0004, two-tailed Student’s* t* test. **D** Schematic showing the timing of FWMI induction. **E** Distribution of oligodendrocytes (MBP+) and immature oligodendrocytes (DM20+) at 5 and 10 dpi in FWMI (scale bars, 50 μm). **F** Relative proportion of MBP density in control and LCN2^*KD*^ mice at 5 and 10 dpi in FWMI (*n =* 3 fields per group). *P <0.05, ***P <0.001 *vs* control; ^###^*P <*0.001 *vs* 5 dpi, two-way ANOVA with Sidak's multiple comparisons test. **G** Image of OPCs (O4+) and oligodendrocytes (CNPase+) in cultured OPCs treated with vehicle or 40 ng/μL LCN2 along with T3 for 24 h (scale bar, 25 μm). **H** Analysis of the CNPase fluorescence intensity after vehicle or LCN2 treatment (*n =* 4 fields per group; ***P = 0.0008, two-tailed Student’s* t* test).

We further investigated whether LCN2 regulated myelin membrane compaction/maturation. During the remyelination phases of SAH or MS, the prominent enlargement of the inner myelin tongues of LCN2^*KD*^ mice was associated with small-diameter axons (0.4 < diameter < 1; measurement protocol outlined in Fig. S3A, B, E). The persistent enlargement of inner tongues may thus result either from an increased membrane growth rate or impaired actin disassembly and myelin membrane compaction [31]. We first measured the thickness of compacted layers to assess these hypotheses because an increased membrane growth rate would result in a thicker myelin sheath. Plotting myelin thickness against the axon diameter in LCN2^*KD*^ mice revealed that the myelin thickness was most prominently increased in small axons, thereby implying increased membrane growth in early remyelination (Fig. S3C, D). We then assessed the expression of MBP, which is required for actin disassembly and myelin membrane compaction [36]. LCN2^*KD*^ mice showed a significantly increased MBP intensity compared to mice treated with the scrambled controls (Fig. 3F, G); NF200+ myelin sheaths devoid of MBP were also observed in these mice (Fig. 3H), indicating non-compact myelin, as NF200 is normally excluded from compact myelin sheaths by MBP [37]. Finally, we showed that the thicker myelin in LCN2^*KD*^ mice was compact (Fig. 2J).

Overall, these data indicate that remyelination is increased in the brains of LCN2^*KD*^ mice and suggest that high levels of myelin are produced by LCN2-knockdown oligodendrocytes during the recovery phase in the SAH and MS models.

### Inhibition of LCN2 does not Reduce Glial Activation and Inflammation

Some studies have revealed that LCN2 damages the brain by promoting glial activation and the release of pro-inflammatory factors [38, 39]. Therefore, we asked whether the positive effect of LCN2 knockdown on remyelination was due to glial activation and inflammatory injury. We evaluated glial activation at 7 days after SAH by immunofluorescence staining and did not find a significant reduction in the number of microglia and astrocytes in the white matter between control and LCN2^*KD*^ mice (Fig. S4A–C). Furthermore, we used qPCR to assess the expression of the main pro-inflammatory factors IL-1β, TNF-αα, IFN-γ, and Ccl2 in brain tissue. LCN2^*KD*^ mice did not show a significant reduction in the mRNA expression levels of these factors (Fig. S4D–G).

### LCN2 Inhibits OPC Differentiation

We next sought to determine whether the cellular mechanism underlying remyelination in LCN2^*KD*^ mice involves oligodendroglial responses. At 14 days after SAH, when remyelination occurred, the number of NG2-positive OPCs was lower in the WM of LCN2^*KD*^ mice than in mice treated with scrambled control (Fig. 3A, B). In contrast, the number of oligodendrocytes (CNPase+) was significantly increased (Fig. 3A, B). Based on these data, LCN2 knockdown promotes OPC differentiation into oligodendrocytes after SAH.

We analyzed the proportion of Olig2+ CC1+ cells that were mature oligodendrocytes and Olig2+ CC1− cells that were immature cells to specifically assess this hypothesis and found a higher proportion of mature oligodendrocytes in the WM of LCN2^*KD*^ mice (Fig. 3C). Although LCN2 knockdown increased the number of Olig2+ CC1+ cells in the WM after SAH, the total number of Olig2+ cells was not significantly altered, suggesting that LCN2^*KD*^ mice show accelerated differentiation into mature oligodendrocytes but no change in OPC proliferation.

In addition to whole-brain demyelinating diseases, we sought to determine whether the efficient remyelination process differed in a focal WMI model established in LCN2^*KD*^ mice. Using temporally discrete OPC responses following focal demyelinating WM injury (FWMI) (Fig. 3D), the proportions of mature oligodendrocytes (MBP+) and immature oligodendrocytes (DM20+) were assessed at the time of initiation of oligodendrocyte differentiation (5 dpi) and remyelination (10 dpi) (Fig. 3E). LCN2^*KD*^ mice displayed a higher proportion of mature oligodendrocytes (MBP+) after FWMI. Thus, LCN2 inhibition also enhances oligodendrocyte differentiation following FWMI (Fig. 3F).

We first assessed OPC proliferation after SAH induction and LCN2 treatment to directly assess the effects of LCN2 on OPC behavior. After counting the number of OPCs (NG2+ Olig2+) at 1, 3, 7, 14, and 28 days after SAH, we did not find a significant difference in OPC proliferation in LCN2^*KD*^ mice (Fig. S5A, B). In addition, a gradient concentration of LCN2 was used to directly treat cultured OPCs, and no significant changes in viability were detected using CCK-8 in cells treated with most concentrations of LCN2; high concentrations (160 ng/µL) of LCN2 reduced viability measured with the CCK-8 assay, probably because of the cytotoxicity associated with high concentrations (Fig. S5C). As brain injury-induced increases in LCN2 expression are unlikely to reach such high concentrations [40], these data exclude an effect of LCN2 on OPC proliferation.

We subsequently assessed the effect of LCN2 on OPC migration using a cell wound-healing assay. After 24 h, the mean migration distance of the 40 ng/µL LCN2 treatment group was not significantly different from that of the LCN2-null treatment group (Fig. S5D, E). Apart from the high concentration of LCN2, most concentrations did not affect the average migration rate of OPCs compared with the LCN2-null treatment group, suggesting that LCN2 does not affect OPC migration (Fig. S5F).

Finally, we took advantage of T3 to induce OPC differentiation and assessed the effect of LCN2 on this process. Treatment with 40 ng/µL LCN2 abolished the T3-induced differentiation of pre-oligodendrocytes (O4+CNPase–) into oligodendrocytes (O4+CNPase+), suggesting an inhibitory effect of LCN2 on OPC differentiation (Fig. 3G, H).

### LCN2 Activates the SLC22A17/EGR1 Pathway in OPCs

**Fig. 4:**
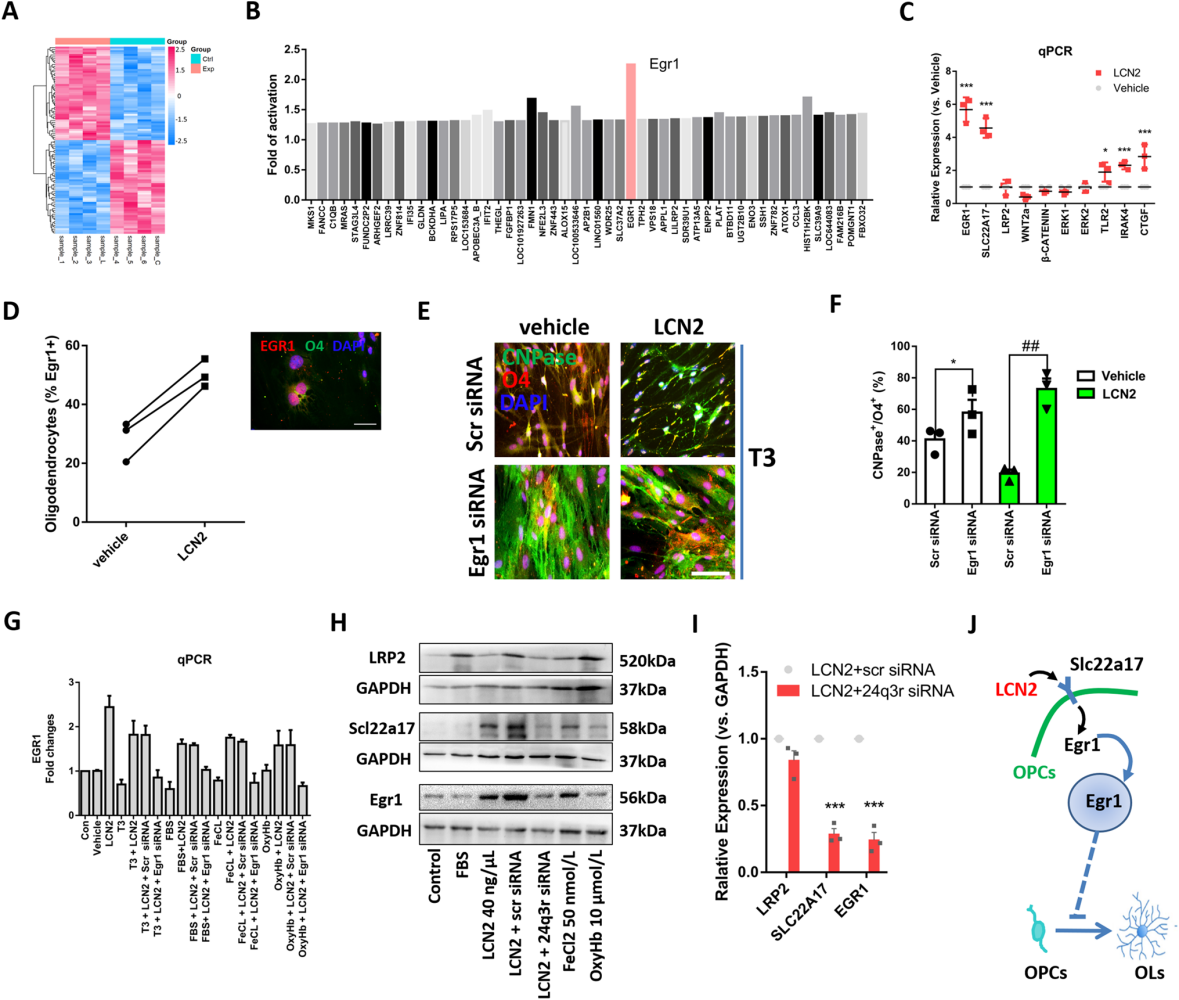
LCN2 activates the SLC22A17/EGR1 pathway in OPCs. **A** Cluster map of differentially-expressed genes in the vehicle (Ctrl) and LCN2 (Exp) groups. Fold changes in 100 differentially-expressed genes ranging from −1.5 to 1.5. **B** Mean fold activation of some differentially-expressed genes in OPCs treated with LCN2 compared with vehicle. EGR1 shows significant activation (*P =* 0.00015,* t* = 5.25, df = 6, Limma moderated* t* test; *n =* 4 independent RNA preparations and analyses). The complete list of regulated genes is reported in Supplementary Table 1. **C** qPCR verification of the relative expression of some differentially-expressed genes in OPCs treated with LCN2 compared with vehicle (*P <0.05, ***P <0.001 *vs* control group, one-way ANOVA with Dunnett's multiple comparisons test). **D** Left panel, analysis of EGR1-positive OPCs (O4+) after treatment with vehicle or LCN2 (*P = 0.0145, two-tailed Student’s* t* test. Right panel, co-localization of EGR1 and O4 in OPC cultures. **E** Images of OPCs (O4+) and oligodendrocytes (CNPase+) in cultured OPCs following scr siRNA or EGR1 siRNA treatment after T3 induction for 24 h (scale bar, 25 μm). **F** Proportions of O4+CNPase+ cells among total OPCs following scr siRNA or EGR1 siRNA treatment after T3 induction for 24 h (*P <0.05, ^##^*P <* 0.01 *vs* scr siRNA, two-way ANOVA with Sidak's multiple comparisons test). **G** qPCR analysis of the fold change in EGR1 expression in cultured OPCs after treatment with vehicle, T3, LCN2, FBS, FeCl2, OxyHb, scr siRNA, EGR1 siRNA, or the respective combinations (*P <0.05, ^##^*P <*0.01, two-way ANOVA with Sidak's multiple comparisons test). **H** Western blots showing the levels of LRP2 (520 kDa), SLC22A17 (58 kDa), EGR1 (56 kDa), and the reference protein GAPDH (37 kDa) after treatment with vehicle, LCN2, FBS, FeCl2, OxyHb, scr siRNA, 24q3r (SLC22A17) siRNA, or their respective combinations. **I** Relative expression of LRP2, SLC22A17, and EGR1 in OPCs treated with LCN2 plus scr siRNA or 24q3r (SLC22A17) siRNA (***P <0.001, two-way ANOVA with Sidak's multiple comparisons test). **J** Schematic of the signaling pathway potentially activated by LCN2 that is involved in OPC differentiation. LCN2 increases the expression of its transmembrane receptor SLC22A17 on OPCs and activates the transcription factor EGR1, thereby inhibiting the differentiation of OPCs into oligodendrocytes (OLs).

Based on the data described above, LCN2 is a negative regulatory factor for OPC differentiation after WMI. We next sought to explore the molecular mechanisms underlying OPC differentiation. The activation and inactivation of molecular pathways in LCN2-stimulated oligodendroglial lineage cells for 24 h was simultaneously assessed by measuring the mRNA levels of signaling proteins using an Affymetrix GeneChip Human Gene 1.0ST Array. Differentially-expressed genes were normalized to GAPDH expression (the complete list of regulated genes is reported in Supplementary Table 1), and values were then normalized to the vehicle control group (Fig. 4A).

We found that LCN2 reduced the expression of STRBP, DYNC1I2, FMO8P, API5, and ZFN525 but increased the expression of PLAT, NFE2L3, IFIT2, FMN1, and SLC39A9, as verified by RT–PCR (Fig. S6). Regarding OPC differentiation, LCN2 did not significantly activate the typical LINGO-1, Notch, ErbB2, PXRγ, and histamine receptors 1/3 that modulate oligodendrocyte differentiation. However, LCN2 slightly decreased the expression of genes involved in positive regulatory signaling, including Wnt/β-catenin, Sema4G/plexin, and ERK1/2, while increasing the expression of genes involved in negative regulatory signaling, such as CTGF and TLR2/IRAK4 (Fig. S7A). LCN2 did not activate some reported inhibitory factors of oligodendroglial differentiation, but the upregulation of RhoA was ascribed to the LCN2-induced stress response (Fig. S7B).

Notably, LCN2 caused marked activation of the transcription factor EGR1, which was verified by qPCR and immunostaining (Fig. 4B–D). In fact, increased numbers of EGR1-positive OPCs and levels of EGR1 expression were found after SAH (Fig. S8A–C). Therefore, the activation of EGR1 may mediate LCN2-regulated remyelination by inhibiting OPC differentiation. We used an siRNA to suppress the EGR1 activation that accompanied LCN2 treatment to test this hypothesis. Double-labeling of CNPase and O4 showed that the inhibitory effect of LCN2 on the differentiation of OPCs (O4+CNPase–) into oligodendrocytes (O4+CNPase+) was neutralized in the EGR1 siRNA group compared with the scr siRNA group (Fig. 4E). EGR1 siRNA also promoted T3-induced OPC differentiation (Fig. 4F). Nevertheless, treatment with FBS promoted OPC differentiation, while FeCl_2_ and OxyHb inhibited OPC differentiation and even reduced EGR1 mRNA levels (Fig. 4G).

In addition, LCN2 increased the expression of its receptor SLC22A17 (also named 24q3r) but did not increase LRP2 expression (Fig. 4C). We next verified that LCN2 and its receptors regulated EGR1 activation using western blotting. Treatment with LCN2 for 24 h increased SLC22A17 and EGR1 but not LRP2 levels in cultured OPCs (Fig. 4H). This effect of LCN2 on the activation of SLC22A17 and EGR1 was reversed by the administration of 24q3r siRNA (Fig. 4I). FeCl_2_ had similar effects on the activation of SLC22A17 and EGR1, suggesting that iron transport may be associated with OPC differentiation [41]. However, OxyHb, which is responsible for iron transport and metabolism through proteins such as LCN2, did not induce significant changes in the activation of SLC22A17 and EGR1 in OPCs. FBS did not increase the expression of SLC22A17 and EGR1, but upregulated LRP2 expression.

Taken together, these data indicate that LCN2 activates SLC22A17/EGR1 signaling but not LRP2, thereby inhibiting the differentiation of OPCs into oligodendrocytes (Fig. 4J).

### Inactivation of EGR1 Promotes OPC Differentiation in Developing and Injured Brains

EGR1 has various functions in fibroblasts, epithelial cells, lymphocytes, microvascular endothelial cells, and neurons, according to previous studies [42]. We precisely determined whether EGR1 activation was required for the inhibitory effect of LCN2 on OPC differentiation by constructing a constitutive conditional knockout in which oligodendroglial lineage cells were unable to respond to LCN2 due to EGR1 excision in OPCs (PDGFRa-Cre; EGR1^fl/fl^, CKO) (Fig. S9A). Tamoxifen effectively reduced the EGR1 mRNA level in the whole brains of CKO mice (Fig. S9B). According to immunofluorescence staining, EGR1 expression was confirmed to be eliminated in PDGFRα+ oligodendrocyte lineage cells from tamoxifen-treated CKO mice (Fig. S9C, D). However, the conditional knockout of EGR1 in OPCs did not affect the number of OPCs (PDGFRα+) in adult mouse brains (Fig. S9E, F). CKO mice displayed no abnormal behaviors, indicating a lack of evident myelin damage.

**Fig. 5:**
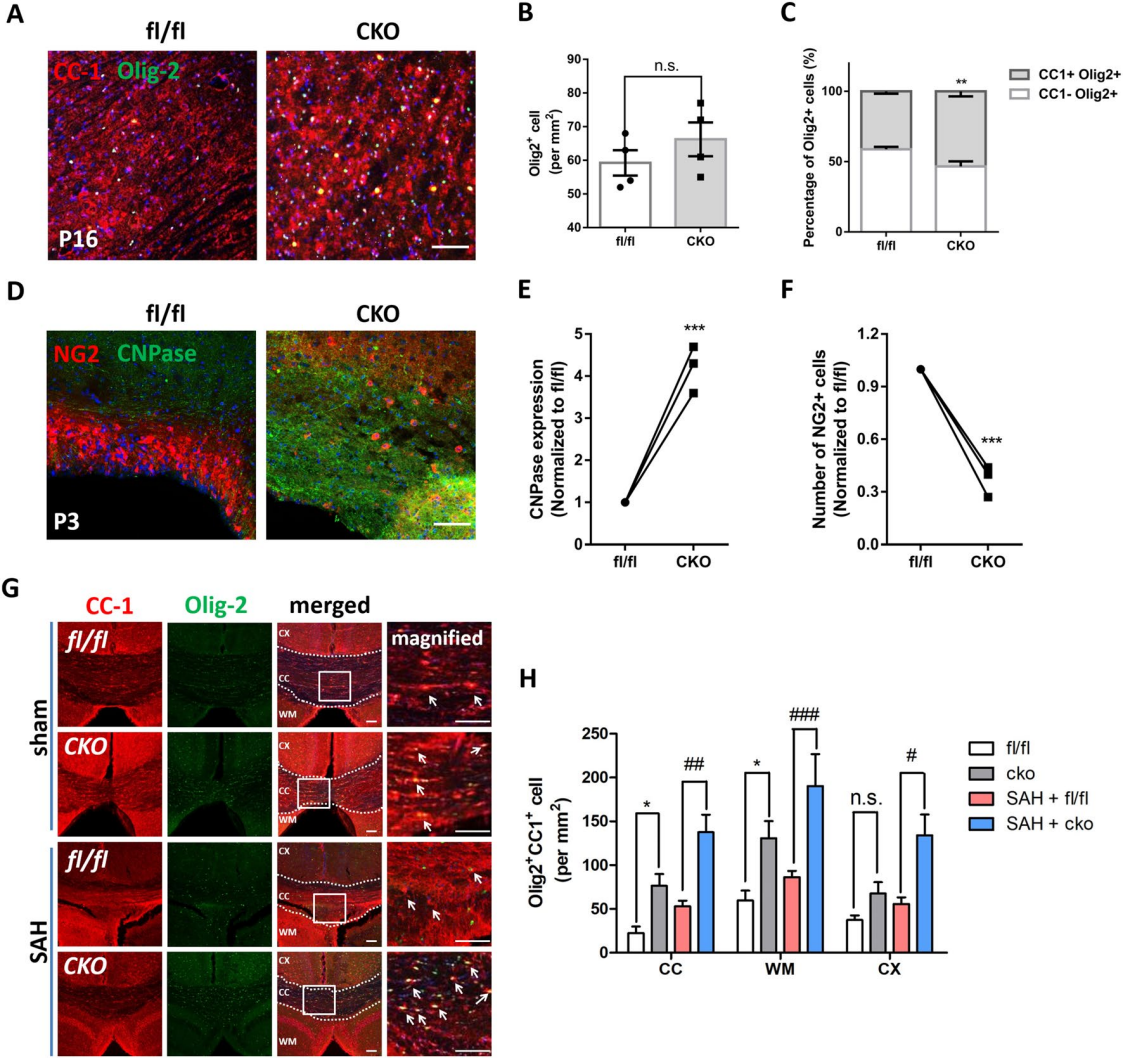
Inactivation of EGR1 promotes OPC differentiation in developing and injured brains. **A** Images of mature oligodendrocytes (CC1+Olig2+) in fl/fl and CKO mice at P16 (scale bar, 75 μm). **B** Total number of Olig2+ cells in EGR1^fl/fl^ and CKO mice at P16 (*n =* 4 fields per group; *P =* 0.9609, two-tailed Student’s* t* test). **C** Percentages of CC1+ and CC1– cells among all Olig2+ cells in fl/fl and CKO mice at P16 (*n =* 4 fields per group; **P = 0.0074, multiple* t* test). **D** Images of OPCs (NG2+) and oligodendrocytes (CNPase+) in the subventricular zone of fl/fl and CKO mice at P3. Scale bar, 75 μm. **E** Normalized CNPase expression in EGR1^fl/fl^ and CKO mice at P3. Two-tailed Student’s* t* test, ***P = 0.0005. **F** Normalized NG2 expression in EGR1^fl/fl^ and CKO mice at P3. Two-tailed Student’s* t* test, ***P = 0.0002. **G** Image of mature oligodendrocytes (CC1+Olig2+) in EGR1^fl/fl^ and CKO mice at 7 days after sham or SAH surgery, and a magnified view of white frame in the CC is shown. Scale bar, 75 μm. **H** Analysis of CC1+Olig2+ cells in different brain regions (CC, WM, and CTX) of EGR1^fl/fl^ and CKO mice at 7 days after sham or SAH surgery. Two-way ANOVA with Sidak's multiple comparisons test: *P < 0.05 compared with the sham + EGR1^fl/fl^ group; ^#^*P <* 0.05, ^##^*P <* 0.01, and ^###^*P <* 0.001 compared with the SAH + EGR1^fl/fl^ group.

We next assessed whether CKO affects the development of myelination in mice. At P16, when myelination is normally under way, the number of oligodendrocyte lineage cells (Olig2+) was slightly increased in CKO mice; CKO mice had more mature OPCs (CC1+Olig2+) than EGR1^fl/fl^ mice (Fig. 5A–C). The increase in oligodendrocyte maturation in EGR1 CKO mice was found as early as P3 during development. More oligodendrocytes (CNPase+) were observed in the subventricular zone, where OPC proliferation and myelin repair occur (Fig. 5D–F). CKO reduced the number of OPCs (NG2+), suggesting that the inactivation of EGR1 in OPCs facilitates myelination in the developing brain.

The CKO mice were subjected to SAH induction to further analyze whether CKO affected OPC differentiation under pathological conditions (Fig. S10A). CKO eliminated the increase in EGR1 expression in OPCs after SAH (Fig. S10B, C). Then, we applied immunofluorescence staining to assess oligodendrocyte maturation at 14 days after SAH. In the sham groups, CKO increased the proportion of mature oligodendrocytes (CC1+Olig2+) in the CC and WM; in the SAH groups, this proportion was higher in CKO mice than in EGR1^fl/fl^ mice (Fig. 5G, H).

Overall, these data revealed that the inactivation of EGR1 promotes OPC differentiation during development and injury.

### Specific Inactivation of EGR1 Promotes Remyelination and Recovery of White Matter Function

We have shown that the specific inactivation of EGR1 promotes OPC differentiation; thus, the question of whether EGR1 promotes the recovery of WM function should be answered.

At 10 dpi, the number of NG2-positive OPCs located in WM lesions was lower in CKO mice than in EGR1^fl/fl^ mice; moreover, the normalized expression of CNPase was significantly increased in CKO mice, indicating that the inactivation of EGR1 promoted remyelination after FWMI (Fig. S11A–C). During the myelin recovery phase of the MS model (5 wpi), the double-labeling results showed that remyelinated MAG expression was increased in the spinal cord of CKO mice; furthermore, a thicker myelin sheath surrounding almost all axon diameters was observed in the WM of CKO mice than in EGR1^fl/fl^ mice (Fig. S11D–F). We performed BMS scoring and a water maze test to examine WM function and the behavior of CKO mice during remyelination. Compared with EGR1^fl/fl^ mice, the BMS score, which represents the motor function of CKO mice, was increased from 4 to 6 wpi (Fig. S11G). The swimming distance and escape latency were reduced in CKO mice compared with EGR1^fl/fl^ mice (Fig. S11H, I). Therefore, the inactivation of EGR1 in OPCs promotes remyelination and behavioral recovery in FMWI and MS models.

**Fig. 6:**
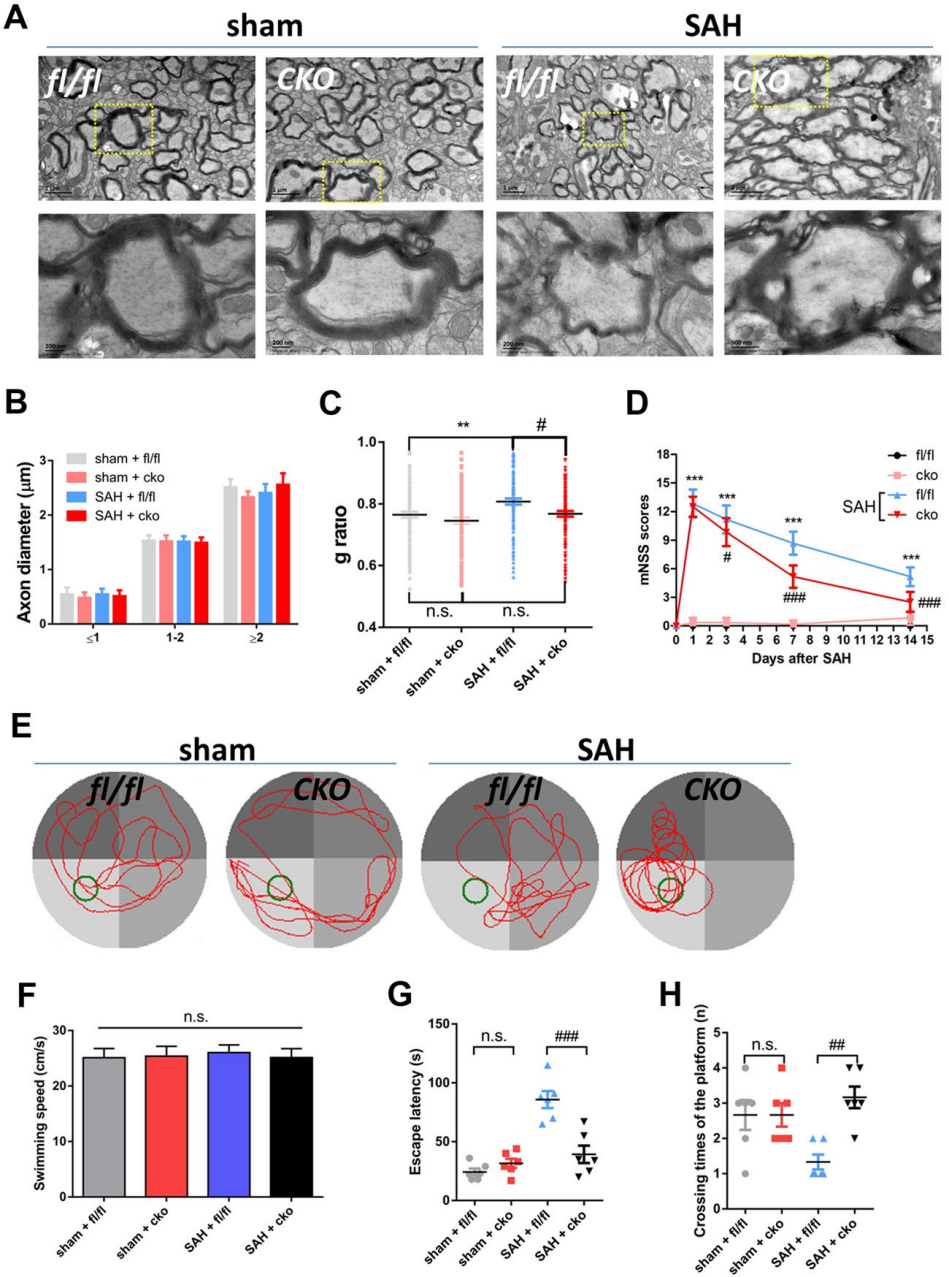
Specific inactivation of EGR1 promotes remyelination and recovery of white matter function. **A** Ultrastructure of the WM of EGR1^fl/fl^ and CKO mice at 7 days after sham or SAH surgery. The areas bounded by yellow frames are magnified below (scale bars, 2 μm or 200 nm). **B** Axon diameter in EGR1^fl/fl^ and CKO mice at 7 days after sham or SAH surgery (no difference in diameter <1, 1–2, and <2, two-way ANOVA with Sidak's multiple comparisons test). **C** G-ratio of EGR1^fl/fl^ and CKO mice at 7 days after sham or SAH surgery (**P <0.01, ^#^*P <*0.05 *vs* SAH + EGR1^fl/fl^ group, one-way ANOVA with Dunnett's multiple comparisons test). **D** mNSS evaluation of EGR1^fl/fl^ and CKO mice at 0, 1, 3, 7, and 14 days after sham or SAH surgery (***P <0.001 *vs* sham + EGR1^fl/fl^ group, ^#^*P <*0.05, ^###^*P <*0.001 *vs* SAH + EGR1^fl/fl^ group, two-way ANOVA with Sidak's multiple comparisons test). **E** Swimming trajectory of EGR1^fl/fl^ and CKO mice in the water maze pool. **F** Analysis of the swimming speed of EGR1^fl/fl^ and CKO mice with sham or SAH surgery (n.s. no significant difference, one-way ANOVA with Dunnett's multiple comparisons test). **G** Escape latency before finding the platform of EGR1^fl/fl^ and CKO mice with sham or SAH surgery (n.s. no significant difference *vs* sham + EGR1^fl/fl^ group; ^###^*P =* 0.0002 *vs* SAH + EGR1^fl/fl^ group, one-way ANOVA with Dunnett's multiple comparisons test). **H** Number of platform crossings within 120 s by EGR1^fl/fl^ and CKO mice with sham or SAH surgery.. n.s. no significant difference *vs* sham + EGR1^fl/fl^ group; ^##^*P =* 0.0078 *vs* SAH + EGR1^fl/fl^ group, one-way ANOVA with Dunnett's multiple comparisons test.

Remyelination was analyzed 14 days after SAH using electron microscopy (Fig. 6A). The axon diameter in EGR1^fl/fl^ mice was the same as that in CKO mice at all scales (Fig. 6B). In the sham groups, no significant difference in the g-ratio was found between CKO and EGR1^fl/fl^ mice. After SAH, the thickness of myelin was increased in the CKO group compared to the EGR1^fl/fl^ group (Fig. 6C). In the behavioral assessment, CKO did not significantly change the mNSS scores of the sham groups. However, compared to EGR1^fl/fl^ mice, CKO mice showed decreased mNSS scores at 3, 7, and 14 days after SAH (Fig. 6D). We next used the Morris water maze to assess cognitive recovery after SAH (Fig. 6E). No significant difference in swimming speed was found between the sham and SAH groups. In the sham group, CKO mice showed no significant differences in escape latency, platform crossings, or swimming distance from EGR1^fl/fl^ mice. In the SAH group, CKO exhibited a shorter swimming distance and escape latency and a reduced number of platform crossings (Fig. 6F–H).

In summary, these data indicate that the specific inactivation of EGR1 in OPCs promotes remyelination, thereby providing a promising target for the functional recovery of WM.

## Discussion

We investigated the function of LCN2 and the possible molecular mechanism in WMI induced by SAH (atypical and acute) and MS (typical and chronic) to identify therapeutic targets for remyelination and WMI after SAH and other diseases. Using whole-transcript expression microarrays, conditional knockout, *in vitro* manipulation of oligodendrocyte lineage cells, and *ex vivo*/*in vivo* modeling of remyelination, we elucidated the inhibitory effects of LCN2 and activated SCL22A17/EGR1 signaling on appropriate oligodendrocyte differentiation in WMI. These findings extend previous studies that did not report an effect of LCN2 on myelin [38, 43] by documenting a direct and disease-relevant role in oligodendrocyte lineage cells. Our results also extend the list of negative regulators of oligodendroglial differentiation to now include the regulation of myelin repair and the appropriate response to WMI in human disease [6].

Resident OPCs are the main promoters of remyelination after CNS injury and eventually promote partial clinical remission [44]. However, the understanding of the efficiency of oligodendrogenesis and remyelination in the adult CNS is limited [45] for two complementary reasons. First, OPC reservoirs in the brain appear to be depleted and rarely migrate into lesions, especially during the progressive phase of the disease, but we detected excess OPCs in the WM after SAH, consistent with other findings from MS and intracranial hemorrhage models [46]. Second, and most likely of greater relevance, inhibitors of oligodendroglial differentiation and maturation may act either alone or synergistically to interfere with regeneration processes [47]. Inhibitory components, such as LINGO-1, Id2, Id4, and IFN-γ, have been identified over the past few years [6].

LCN2, also known as NGAL, 24P3, and SIP24, is an acute secreted protein that belongs to the lipocalin protein family and regulates various pathophysiological processes in the immune response, iron metabolism, lipid metabolism, and cell proliferation and differentiation [48]. After central nervous system injury, LCN2 expression is significantly increased and it exerts paracrine and autocrine effects on damaged brain tissue [34]. Zhou and colleagues found that astrocyte-derived LCN2 regulates pathological phagocytosis, leading to neural injury [49]. Therefore, LCN2 might represent a biomarker of acute brain injury [10, 11] and a “help-me” signal for repair by inducing reactive glial cells and the transformation of murine vascular cells into repair phenotypes [12]. LCN2^-/-^ mice exhibit significantly lower T2 hypersignals in the CC after SAH, suggesting less acute WMI [8]. In addition, Nam *et al.* found that the degree of demyelination in LCN2^-/-^ mice with autoimmune encephalomyelitis is significantly reduced compared with that in wild-type mice [38]. Al Nimer *et al*. reported a significant increase in LCN2 expression in patients with progressive MS, and LCN2 levels were several times higher in the brain tissue than that in cerebrospinal fluid, suggesting that LCN2 levels are closely associated with WM demyelination [9]. In the present study, LCN2 was present at particularly high levels in WM lesions and negatively regulated remyelination. Furthermore, we revealed that LCN2 inhibited OPC differentiation, indicating that LCN2 activation is a novel oligodendroglial differentiation inhibitor. Although problems with oligodendroglial differentiation are a major cause of remyelination failure, a differentiation blockade may also result from a lack of differentiation-inducing signals [50]. In the present study, we used the thyroid hormone T3, which enhances oligodendroglial differentiation and proliferation early in development [51], to verify the role of LCN2 in the differentiation process. LCN2 inhibits T3-induced oligodendroglial differentiation, raising the question of how LCN2 affects T3 signaling. The increased enzymatic deactivation of T3 may play a role in impaired remyelination in experimental autoimmune encephalomyelitis [52]. Thus, further studies are needed to determine whether T3 signaling is still involved in the progression of remyelination in models of SAH or MS. Overall, these results provide new putative targets for future remyelination therapies.

Numerous experimental and clinical studies have shown that LCN2 is upregulated during acute and chronic injury [53]. However, researchers are still disputing whether LCN2 exerts protective or deleterious effects after injury [34]. The discrepancy is associated with the controversial role of LCN2 in the inflammatory response. In hepatology studies, LCN2-null mice showed a significantly lower recruitment of neutrophils and leukocytes, indicative of protective effects, suggesting that LCN2 might act as an intrinsic “help-me” sensor upon injury [54]. In the brain, LCN2 promotes neuroinflammation but appears to exert neuroprotective effects on some diseases [55]. In fact, microarrays have shown that LCN2 actually induced an inflammatory response in OPCs when a group of upregulated inflammatory factors was examined after LCN2 stimulation. However, LCN2 knockdown is not sufficient to alleviate MS- or SAH-induced neuroinflammation because the inhibition of LCN2 failed to repress the activation of microglia and astrocytes in the two disease models. A parallel mechanism, such as iron dysregulation and neurotoxicity, might induce the neuroinflammatory process [56], or a very low level of LCN2 might sensitively activate the downstream neuroinflammatory reaction [57]. The precise effect of LCN2 on the CNS is far from completely understood and is considered multifaceted. From a clinical perspective, LCN2 could be targeted therapeutically to dampen pro-inflammatory astrocytic activation [58]. Nonetheless, currently, the absence of a specific antagonist for LCN2, as well as the poorly elucidated mechanism by which LCN2 induces astrocytic neurotoxicity by binding to specific receptors, makes the task of counteracting LCN2 effects on the progression of CNS diseases very challenging.

The effects of LCN2 on oligodendrocyte differentiation were mediated by the receptor SCL22A17 and downstream transcription factor EGR1, as its neutralization in OPCs eliminated the LCN2-driven suppression of oligodendrocyte differentiation and hypomyelination. Accordingly, we showed that EGR1 expression in oligodendroglial lineage cells *in vivo* coincided with oligodendrocyte differentiation and myelin generation during development and following injury. During hypomyelination after injury, a concomitant upregulation of EGR1 was found. The concurrent downregulation of several transcription factors, including Id2, EGR1, and Sox11, is critical for OPC differentiation [59], and importantly, the increased expression of these transcription factors impedes oligodendrocyte differentiation and myelination during development and after WMI. In esophageal squamous cell carcinoma, EGR1 is the key transcriptional activator of LCN2 within a positive LCN2-MEK/ERK-LCN2 loop to promote the migration and invasion of esophageal squamous cell carcinoma cells [60]. Thus, the downregulation of EGR1 after injury may enable oligodendroglial lineage cells to respond and undergo successful remyelination.

The differentiation of OPCs into mature oligodendrocytes is also associated with the activation or inactivation of other transcription factors, such as PKCα, TCF4, and NFATc4[44, 61]. Although we did not detect significant changes in the expression of these genes after LCN2 stimulation in cultured OPCs, *in vivo* findings in individuals with MS and other diseases confirmed the nuclear translocation and activation of oligodendrocyte differentiation-dependent transcription factors, suggesting that complementary pathways likely coordinate the oligodendroglial lineage and may also respond in parallel. These LCN2-insensitive and LCN2-sensitive transcription factors may be divided into distinct subsets of OPCs, since a single-cell RNAseq database confirmed distinct expression patterns of the above factors within OPCs [62]. In addition, different transcription factors may be activated by distinct cellular regulatory signals. For example, we found alternative LCN2-induced mechanisms that have previously been shown to modulate oligodendrocyte differentiation, suggesting that an alternate mechanism may be involved in the LCN2-dependent decrease in oligodendrocyte differentiation during remyelination.

Admittedly, some limitations of the present study should also be mentioned. First, we used an siRNA approach to study the role of LCN2 in remyelination after WMI. However, based on the efficiency and toxicity of gene interference, mouse models with LCN2 deletion (LCN2^-/-^) or even conditional LCN2 deletion in the brain (e.g., Nestin^cre/cre^/LCN2^fl/fl^) may be required for further study. Second, recent efforts in high-throughput screening have identified a number of compounds that potently promote OPC differentiation and remyelination [44, 63]; thus, the identification of which pharmacological strategy is feasible and realistic to regulate LCN2 or EGR1 is an issue that should be resolved. Third, additional details of the mechanism by which LCN2 modulates EGR1 activation should be elucidated using genetic and pharmacological approaches.

## Conclusions

In summary, we provide the first evidence to identify the involvement of SCL22A17/EGR1 in LCN2-mediated insufficient OPC remyelination in both typical/chronic WM disorders (MS) and atypical/acute WM disorders (SAH), although these diseases have distinct etiologies. Our data reveal the inhibitory effects of LCN2 on OPC differentiation. Thus, we propose that therapies specifically removing LCN2 or inactivating SCL22A17/EGR1 signaling in oligodendroglial lineage cells might represent a novel strategy to enhance differentiation and remyelination in patients with WMI.

## Supplementary Information

Below is the link to the electronic supplementary material.

Supplementary file1 (PDF 1401 kb)

Supplementary file2 (PDF 202 kb)

Supplementary file3 (XLS 4239 kb)

## Data Availability

All data generated or analyzed during this study are included in this published article and its supplementary information files. The datasets used and/or analyzed during the current study are available from the corresponding author upon reasonable request.

## References

[1] van Tilborg E, de Theije CGM, van Hal M, Wagenaar N, de Vries LS, Benders MJ (2018). Origin and dynamics of oligodendrocytes in the developing brain: Implications for perinatal white matter injury. Glia.

[2] MacDonald RL, Schweizer TA (2017). Spontaneous subarachnoid haemorrhage. Lancet.

[3] Gaastra B, Ewbank F, Tapper W, Bulters D, Galea I (2022). Long-term cognitive outcome following aneurysmal subarachnoid haemorrhage. J Stroke Cerebrovasc Dis.

[4] Lee SJ, Kim MS, Jang SH (2020). White matter abnormalities in spontaneous subarachnoid hemorrhage: A tract-based spatial statistics study. Stroke.

[5] Villoslada P, Martinez-Lapiscina EH (2019). Remyelination: a good neuroprotective strategy for preventing axonal degeneration?. Brain.

[6] Kremer D, Aktas O, Hartung HP, Küry P (2011). The complex world of oligodendroglial differentiation inhibitors. Ann Neurol.

[7] Ru XF, Gao L, Zhou JR, Li Q, Zuo SL, Chen YJ (2021). Secondary white matter injury and therapeutic targets after subarachnoid hemorrhage. Front Neurol.

[8] Peng K, Koduri S, Ye FH, Yang JT, Keep RF, Xi GH (2022). A timeline of oligodendrocyte death and proliferation following experimental subarachnoid hemorrhage. CNS Neurosci Ther.

[9] Al Nimer F, Elliott C, Bergman J, Khademi M, Dring AM, Aeinehband S (2016). Lipocalin-2 is increased in progressive multiple sclerosis and inhibits remyelination. Neurol Neuroimmunol Neuroinflamm.

[10] Suk K (2016). Lipocalin-2 as a therapeutic target for brain injury: An astrocentric perspective. Prog Neurobiol.

[11] Llorens F, Hermann P, Villar-Piqué A, Diaz-Lucena D, Nägga K, Hansson O (2020). Cerebrospinal fluid lipocalin 2 as a novel biomarker for the differential diagnosis of vascular dementia. Nat Commun.

[12] Xing CH, Lo EH (2017). Help-me signaling: Non-cell autonomous mechanisms of neuroprotection and neurorecovery. Prog Neurobiol.

[13] Lei WJ, Zeng H, Feng H, Ru XF, Li Q, Xiao M (2020). Development of an early prediction model for subarachnoid hemorrhage with genetic and signaling pathway analysis. Front Genet.

[14] Dai JX, Bercury KK, Ahrendsen JT, Macklin WB (2015). Olig1 function is required for oligodendrocyte differentiation in the mouse brain. J Neurosci.

[15] Voskuhl RR, Itoh N, Tassoni A, Matsukawa MA, Ren E, Tse V (2019). Gene expression in oligodendrocytes during remyelination reveals cholesterol homeostasis as a therapeutic target in multiple sclerosis. Proc Natl Acad Sci USA.

[16] Meijer DH, Kane MF, Mehta S, Liu HY, Harrington E, Taylor CM (2012). Separated at birth? The functional and molecular divergence of OLIG1 and OLIG2. Nat Rev Neurosci.

[17] Wang XR, Su YX, Li T, Yu GD, Wang YX, Chen XY (2021). Quetiapine promotes oligodendroglial process outgrowth and membrane expansion by orchestrating the effects of Olig1. Glia.

[18] Li Q, Zhao HL, Pan PY, Ru XF, Zuo SL, Qu J (2018). Nexilin regulates oligodendrocyte progenitor cell migration and remyelination and is negatively regulated by protease-activated receptor 1/ras-proximate-1 signaling following subarachnoid hemorrhage. Front Neurol.

[19] Ru XF, Qu J, Li Q, Zhou JR, Huang SN, Li WY (2021). miR-706 alleviates white matter injury via downregulating PKCα/MST1/NF-κB pathway after subarachnoid hemorrhage in mice. Exp Neurol.

[20] Zuo SL, Ge HF, Li Q, Zhang X, Hu R, Hu SL (2017). Artesunate protected blood-brain barrier via sphingosine 1 phosphate receptor 1/phosphatidylinositol 3 kinase pathway after subarachnoid hemorrhage in rats. Mol Neurobiol.

[21] Pan PY, Zhao HL, Zhang X, Li Q, Qu J, Zuo SL (2020). Cyclophilin a signaling induces pericyte-associated blood-brain barrier disruption after subarachnoid hemorrhage. J Neuroinflammation.

[22] Chen YJ, Zhang Y, Tang JJ, Liu F, Hu Q, Luo CX (2015). Norrin protected blood-brain barrier via frizzled-4/β-catenin pathway after subarachnoid hemorrhage in rats. Stroke.

[23] Sugawara T, Ayer R, Jadhav V, Zhang JH (2008). A new grading system evaluating bleeding scale in filament perforation subarachnoid hemorrhage rat model. J Neurosci Methods.

[24] Werneburg S, Fuchs HLS, Albers I, Burkhardt H, Gudi V, Skripuletz T (2017). Polysialylation at early stages of oligodendrocyte differentiation promotes myelin repair. J Neurosci.

[25] Xu X, Gao WW, Cheng SQ, Yin DP, Li F, Wu YG (2017). Anti-inflammatory and immunomodulatory mechanisms of atorvastatin in a murine model of traumatic brain injury. J Neuroinflammation.

[26] Basso DM, Fisher LC, Anderson AJ, Jakeman LB, McTigue DM, Popovich PG (2006). Basso Mouse Scale for locomotion detects differences in recovery after spinal cord injury in five common mouse strains. J Neurotrauma.

[27] Lee HT, Lee KN, Lin HC, Lee TS (2019). Genetic deletion of soluble epoxide hydroxylase causes anxiety-like behaviors in mice. Mol Neurobiol.

[28] Santiago González DA, Cheli VT, Zamora NN, Lama TN, Spreuer V, Murphy GG, *et al*. Conditional deletion of the L-type calcium channel Cav1.2 in NG2-positive cells impairs remyelination in mice. J Neurosci 2017, 37: 10038–10051. 10.1523/JNEUROSCI.1787-17.2017 PMC5647766 28899915

[29] Weil MT, Schulz-Ëberlin G, Mukherjee C, Kuo-Elsner WP, Schäfer I, Müller C (2019). Isolation and culture of oligodendrocytes. Methods Mol Biol.

[30] Li Q, Chen YJ, Li B, Luo CX, Zuo SL, Liu X (2016). Hemoglobin induced NO/cGMP suppression Deteriorate Microcirculation via Pericyte Phenotype Transformation after Subarachnoid Hemorrhage in Rats. Sci Rep.

[31] Dillenburg A, Ireland G, Holloway RK, Davies CL, Evans FL, Swire M (2018). Activin receptors regulate the oligodendrocyte lineage in health and disease. Acta Neuropathol.

[32] Li D, Lei Y, Deng J, Zhou CJ, Zhang Y, Li WJ (2013). Human but not laboratory borna disease virus inhibits proliferation and induces apoptosis in human oligodendrocytes *in vitro*. PLoS One.

[33] Kawashima T, Yashiro M, Kasashima H, Terakawa Y, Uda T, Nakajo K (2019). Oligodendrocytes up-regulate the invasive activity of glioblastoma cells via the angiopoietin-2 signaling pathway. Anticancer Res.

[34] Ferreira AC, Mesquita SD, Sousa JC, Correia-Neves M, Sousa N, Palha JA (2015). From the periphery to the brain: Lipocalin-2, a friend or foe?. Prog Neurobiol.

[35] Khalil M, Renner A, Langkammer C, Enzinger C, Ropele S, Stojakovic T (2016). Cerebrospinal fluid lipocalin 2 in patients with clinically isolated syndromes and early multiple sclerosis. Mult Scler.

[36] Zuchero JB, Fu MM, Sloan SA, Ibrahim A, Olson A, Zaremba A (2015). CNS myelin wrapping is driven by actin disassembly. Dev Cell.

[37] Aggarwal S, Snaidero N, Pähler G, Frey S, Sánchez P, Zweckstetter M (2013). Myelin membrane assembly is driven by a phase transition of myelin basic proteins into a cohesive protein meshwork. PLoS Biol.

[38] Nam Y, Kim JH, Seo M, Kim JH, Jin M, Jeon S (2014). Lipocalin-2 protein deficiency ameliorates experimental autoimmune encephalomyelitis: The pathogenic role of lipocalin-2 in the central nervous system and peripheral lymphoid tissues. J Biol Chem.

[39] Lee S, Jha MK, Suk K (2015). Lipocalin-2 in the inflammatory activation of brain astrocytes. Crit Rev Immunol.

[40] Ranjbar Taklimie F, Gasterich N, Scheld M, Weiskirchen R, Beyer C, Clarner T (2019). Hypoxia induces astrocyte-derived lipocalin-2 in ischemic stroke. Int J Mol Sci.

[41] Morath DJ, Mayer-Pröschel M (2002). Iron deficiency during embryogenesis and consequences for oligodendrocyte generation *in vivo*. Dev Neurosci.

[42] Duclot F, Kabbaj M (2017). The role of early growth response 1 (EGR1) in brain plasticity and neuropsychiatric disorders. Front Behav Neurosci.

[43] Chun BY, Kim JH, Nam Y, Huh MI, Han S, Suk K (2015). Pathological involvement of astrocyte-derived lipocalin-2 in the demyelinating optic neuritis. Invest Ophthalmol Vis Sci.

[44] Kremer D, Göttle P, Hartung HP, Küry P (2016). Pushing forward: Remyelination as the new frontier in CNS diseases. Trends Neurosci.

[45] Chang AS, Tourtellotte WW, Rudick R, Trapp BD (2002). Premyelinating oligodendrocytes in chronic lesions of multiple sclerosis. N Engl J Med.

[46] Miron VE, Kuhlmann T, Antel JP (2011). Cells of the oligodendroglial lineage, myelination, and remyelination. Biochim Biophys Acta.

[47] Bruce CC, Zhao C, Franklin RJM (2010). Remyelination - an effective means of neuroprotection. Horm Behav.

[48] Lim D, Jeong JH, Song J (2021). Lipocalin 2 regulates iron homeostasis, neuroinflammation, and insulin resistance in the brains of patients with dementia: Evidence from the current literature. CNS Neurosci Ther.

[49] Bi FF, Huang C, Tong JB, Qiu G, Huang B, Wu QX (2013). Reactive astrocytes secrete lcn2 to promote neuron death. Proc Natl Acad Sci U S A.

[50] Franklin RJM, Ffrench-Constant C (2008). Remyelination in the CNS: From biology to therapy. Nat Rev Neurosci.

[51] Franco PG, Silvestroff L, Soto EF, Pasquini JM (2008). Thyroid hormones promote differentiation of oligodendrocyte progenitor cells and improve remyelination after cuprizone-induced demyelination. Exp Neurol.

[52] Castelo-Branco G, Stridh P, Guerreiro-Cacais AO, Adzemovic MZ, Falcão AM, Marta M (2014). Acute treatment with valproic acid and l-thyroxine ameliorates clinical signs of experimental autoimmune encephalomyelitis and prevents brain pathology in DA rats. Neurobiol Dis.

[53] Richardson DR (2005). 24p3 and its receptor: Dawn of a new iron age?. Cell.

[54] Asimakopoulou A, Borkham-Kamphorst E, Tacke F, Weiskirchen R (2016). Lipocalin-2 (NGAL/LCN_2_), a “help-me” signal in organ inflammation. Hepatology.

[55] Kang SS, Ren Y, Liu CC, Kurti A, Baker KE, Bu G (2018). Lipocalin-2 protects the brain during inflammatory conditions. Mol Psychiatry.

[56] Dekens DW, Naudé P, Keijser JN, Boerema AS, de Deyn PP, Eisel ULM (2018). Lipocalin 2 contributes to brain iron dysregulation but does not affect cognition, plaque load, and glial activation in the J20 Alzheimer mouse model. J Neuroinflammation.

[57] Bhusal A, Rahman MH, Lee WH, Bae YC, Lee IK, Suk K (2019). Paradoxical role of lipocalin-2 in metabolic disorders and neurological complications. Biochem Pharmacol.

[58] Xing CH, Wang XS, Cheng CJ, Montaner J, Mandeville E, Leung W (2014). Neuronal production of lipocalin-2 as a help-me signal for glial activation. Stroke.

[59] Swiss VA, Nguyen T, Dugas J, Ibrahim A, Barres B, Androulakis IP (2011). Identification of a gene regulatory network necessary for the initiation of oligodendrocyte differentiation. PLoS One.

[60] Zhao Y, Xia QX, Liu Y, Bai WJ, Yao YB, Ding JY (2019). TCF7L2 and EGR1 synergistic activation of transcription of LCN_2_ via an ERK1/2-dependent pathway in esophageal squamous cell carcinoma cells. Cell Signal.

[61] Trimarco A, Forese MG, Alfieri V, Lucente A, Brambilla P, Dina G (2014). Prostaglandin D2 synthase/GPR44: A signaling axis in PNS myelination. Nat Neurosci.

[62] Marques S, Zeisel A, Codeluppi S, van Bruggen D, Falcão AM, Xiao L (2016). Oligodendrocyte heterogeneity in the mouse juvenile and adult central nervous system. Science.

[63] Deshmukh VA, Tardif V, Lyssiotis CA, Green CC, Kerman B, Kim HJ (2013). A regenerative approach to the treatment of multiple sclerosis. Nature.

