# Supplementary material for: Lipocalin-2-Mediated Insufficient Oligodendrocyte Progenitor Cell Remyelination for White Matter Injury After Subarachnoid Hemorrhage via SCL22A17 Receptor/Early Growth Response Protein 1 Signaling
Source: Neurosci Bull. 2022 Jul 7;38(12):1457–75. doi: 10.1007/s12264-022-00906-w (PMC9723020; doi:10.1007/s12264-022-00906-w)
Supplement: Supplementary file 1 — Supplementary file1 (PDF 1401 kb) [file 12264_2022_906_MOESM1_ESM.pdf]

Supplementary Materials

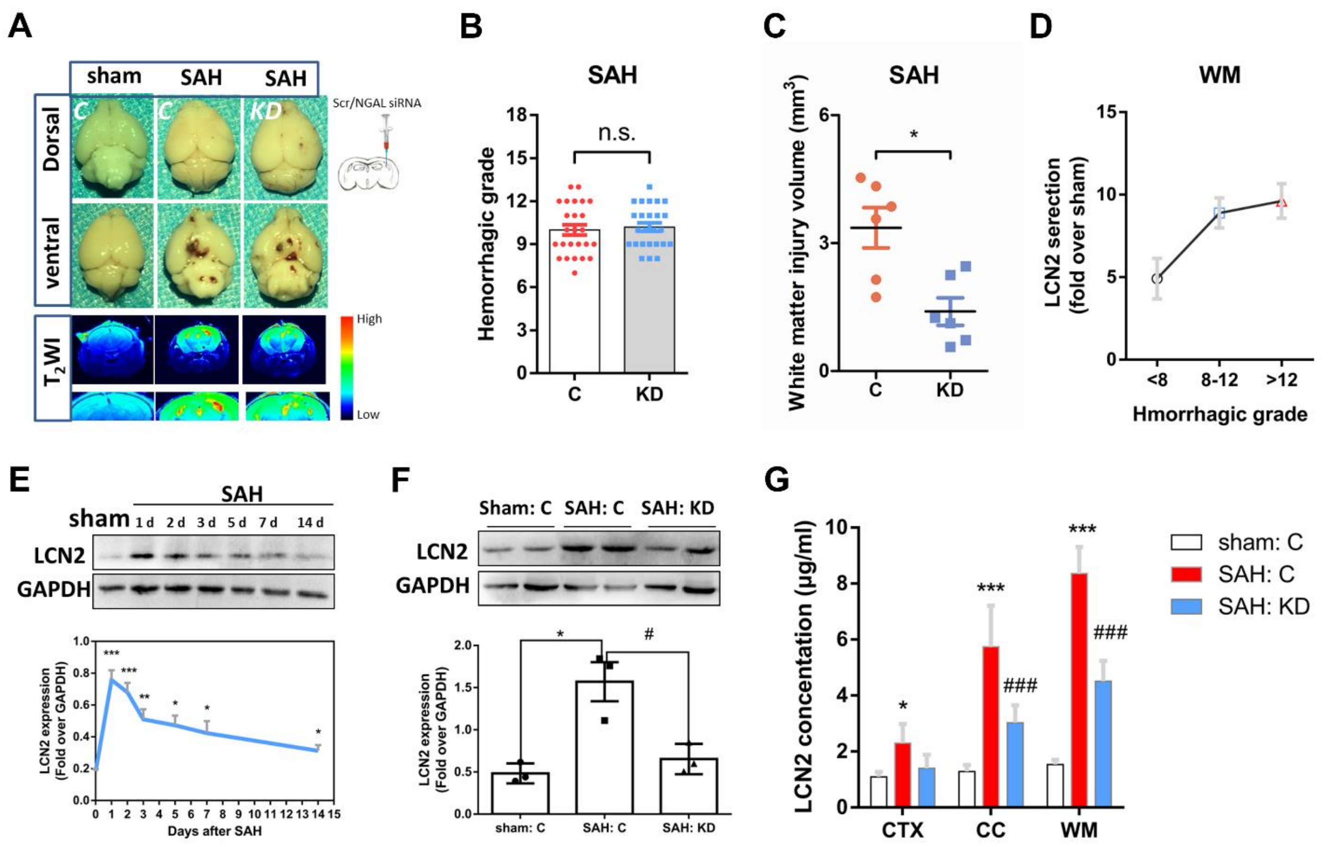

Fig. S1 LCN2 knockdown reduces the accumulation of LCN2 and white matter injury after SAH. **A** Photograph of blood distribution and MRI imaging of T2WI signals in coronal brain sections indicating activin receptor signaling. Right, schematic of the localization of injected siRNA in the brain. **B** Statistics of control (n = 25 mice) and LCN2^{KD} mice (n = 25) at 7 days after SAH. P = 0.558, n.s. no significant difference, two-tailed Student's t test. **C** Scatter plot of WNI volume of control (n = 6 mice) and LCN2^{KD} mice (n = 6) at 7 days after SAH, from T2WI imaging. *P = 0.0285, two-tailed Student's t test. **D** Line chart of LCN2 secretion in brains with different hemorrhagic grades subdivided into <8 (n = 5), 8–12 (n = 8), and >12 (n = 6) groups. **E** Western blots of brain lysates from sham and SAH mice (at 1, 2 3, 5, 7, and 14 dpi) showing the levels of LCN2 (23 kDa), with GAPDH serving as a loading control. The figure below shows the expression of LCN2. *P <0.05, **P <0.01, ***P <0.001, one-way

ANOVA followed by Dunnett's multiple comparisons test. **F** Western blots of brain lysates from control and LCN2^{KD} mice at 7 days after sham or SAH surgery showing the levels of LCN2 (23 kDa), with GAPDH serving as a loading control. The figure below shows the expression of LCN2. n = 3 mice per group. *P = 0.0156 *vs* sham:C group; $^{\#}P$ = 0.0248 *vs* SAH:C group, one-way ANOVA followed by Bonferroni's multiple comparisons test. **G** Statistics of LCN2 concentrations in the WM, CTX, and CC lysates from control and LCN2^{KD} mice at 7 days after sham or SAH surgery. n = 3 mice per group. *P <0.05, ***P <0.001 *vs* sham:C group; $^{\#\#\#}P$ <0.001 *vs* SAH:C group, two-way ANOVA followed by Sidak's multiple comparisons test.

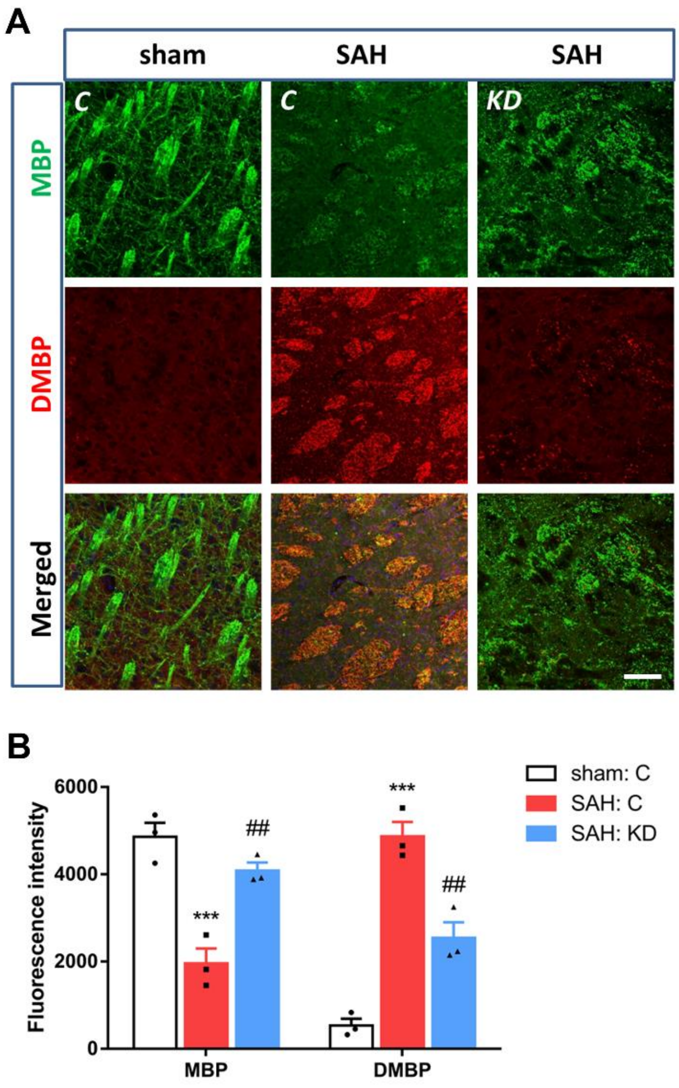

Fig. S2 LCN2 knockdown reduces DMBP expression while increasing MBP expression after white matter injury.

A Images of normal myelin (MBP+) and degraded myelin (DMBP+) in the white matter of control and LCN2^{KD} mice at 7 days after sham or SAH surgery (scale bar, 25 μm). **B** Statistics of the fluorescence intensity of MBP and DMBP in the white matter of control and LCN2^{KD} mice at 7 days after sham or SAH surgery. n = 5 mice per group. ***P <0.001 *vs* sham: **C** group and $^{\#\#}P$ <0.01 *vs* SAH:C group, two-way ANOVA followed by Sidak's multiple comparisons test.

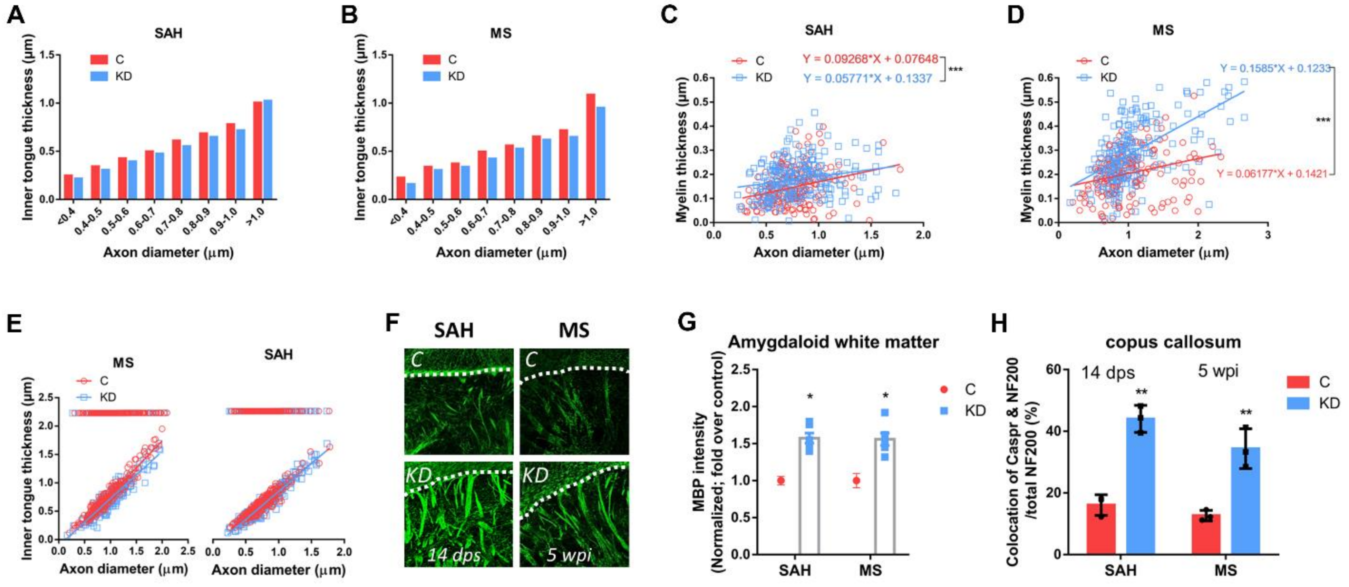

Fig. S3 LCN2 knockdown promotes remyelination after WMI.

A Statistics of mean inner tongue thickness in different axon diameters from the WM of control and LCN2^{KD} mice at 7 days after SAH, from electron microscopic analyses. **B** Statistics of mean inner tongue thickness in different axon diameters from the WM of control and LCN2^{KD} mice at 7 days after MS, from electron microscopic analyses. **C** Myelin thickness *versus* axon diameter in control (magenta) and LCN2^{KD} (green) mice at 7 days after SAH. ***P = 0.0007, extra sum of squares F test between slopes. **D** Myelin thickness *versus* axon diameter in control (magenta) and LCN2^{KD} (green) mice at 7

days after MS. ***P = 0.0001, extra sum of squares F test between slopes. **E** Inner tongue thickness *versus* axon diameter in control (magenta) and LCN2^{KD} (green) mice at 7 days after MS and SAH. The slope lines were obtained using linear regression analysis. **F** Images of normal myelin (MBP+) from the WM of control and LCN2^{KD} mice at 14 days after SAH (dps) and at 5 weeks after MS induction (wpi). The area under the white dotted line indicates the WM. Scale bar, 25 μm. **G** Normalized MBP intensity in control and LCN2^{KD} mice after SAH and MS. *P = 0.0226 (SAH) and *P = 0.0381 (MS), multiple t test. **H** Co-localization of Caspr and NF200 in the corpus callosum of control and LCN2^{KD} mice at 14 days after SAH (dps) and at 5 weeks after MS induction (wpi). The upper panels show images of Caspr and NF200 staining in the corpus callosum of LCN2^{KD} mice after SAH and MS. **P = 0.0015 (SAH) and **P = 0.0083 (MS), multiple t test.

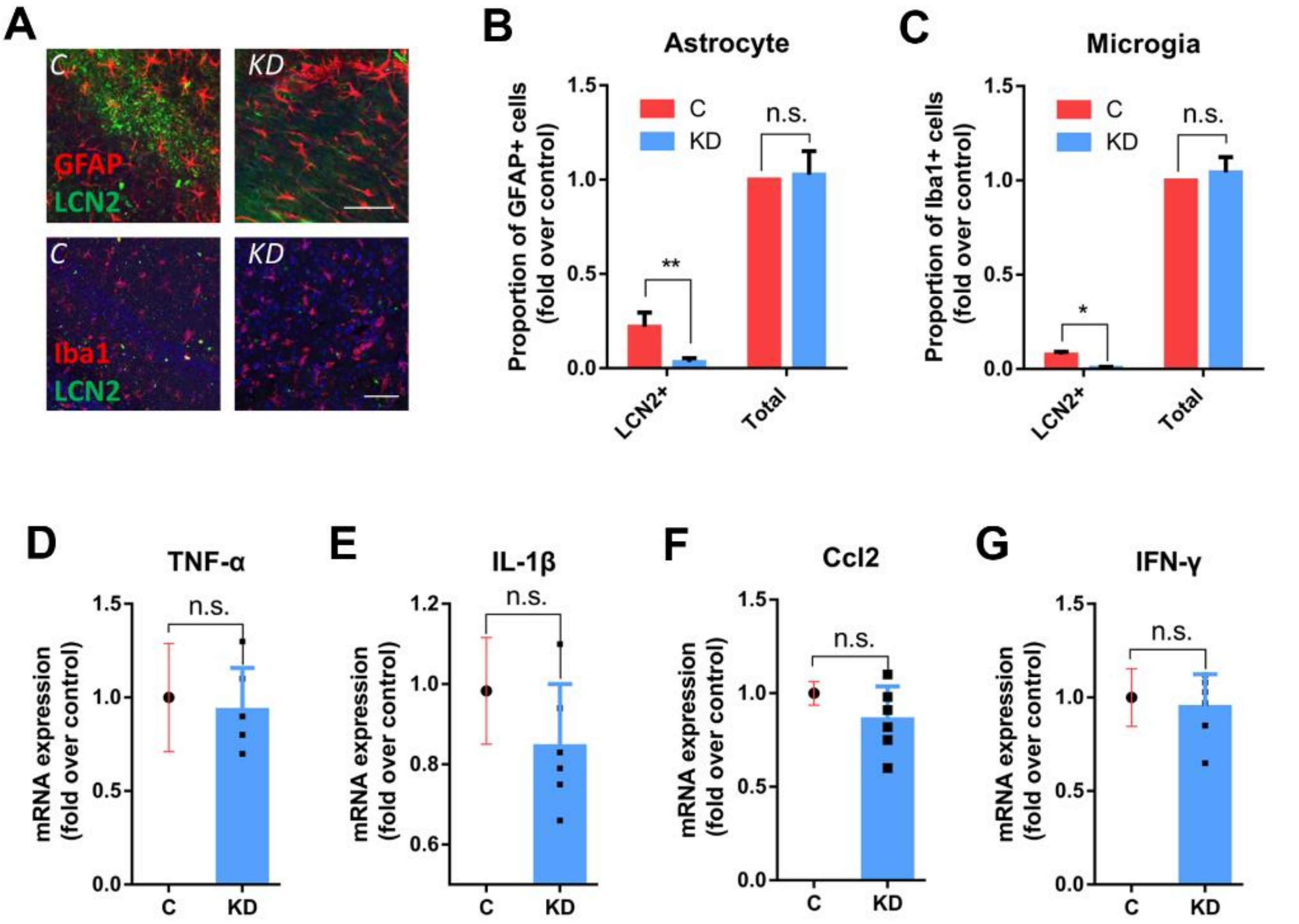

Fig. S4 Inhibition of LCN2 does not reduce glial activation or inflammation.

A Upper panel, images of astrocytes ($GFAP^+$) and LCN2 in the WM of control and $LCN2^{KD}$ mice at 7 days after SAH; lower panel, images of microglia ($Iba1^+$) and LCN2 in the WM of control and $LCN2^{KD}$ mice at 7 days after SAH. Scale bars, 25 µm. **B** Proportions of $GFAP^+$ cells (astrocytes) among $LCN2^+$ cells and total cells ($DAPI^+$) in control and $LCN2^{KD}$ mice at 7 days after SAH. ***P* = 0.0048 for $LCN2^+$ cells, *P* = 0.6724 for total cells; n.s. no significant difference, multiple *t* test. **C** Proportions of $Iba1^+$ cells (microglia) among $LCN2^+$ cells and total cells ($DAPI^+$) from control and $LCN2^{KD}$ mice at 7 days after SAH. **P* = 0.0274 for $LCN2^+$ cells, *P* = 0.4435 for total cells; n.s. no significant difference, multiple *t* test. **D** qPCR of TNF-α mRNA expression in brain extracts from control and $LCN2^{KD}$ mice at 7 days after SAH. *P* = 0.7121; n.s. no significant difference, two-tailed Student's *t* test. **E** qPCR of IL-1β mRNA expression in brain extracts from control and $LCN2^{KD}$ mice at 7 days after SAH. *P* = 0.2115; n.s. no significant difference, two-tailed Student's *t* test. **F** qPCR of CCL-2 mRNA expression in brain extracts from control and $LCN2^{KD}$ mice at 7 days after SAH. *P* = 0.0991; n.s. no significant difference, two-tailed Student's *t* test. **G** qPCR of IFN-γ mRNA expression in brain extracts from control and $LCN2^{KD}$ mice at 7 days after SAH. *P* = 0.5540; n.s. no significant difference, two-tailed Student's *t* test.

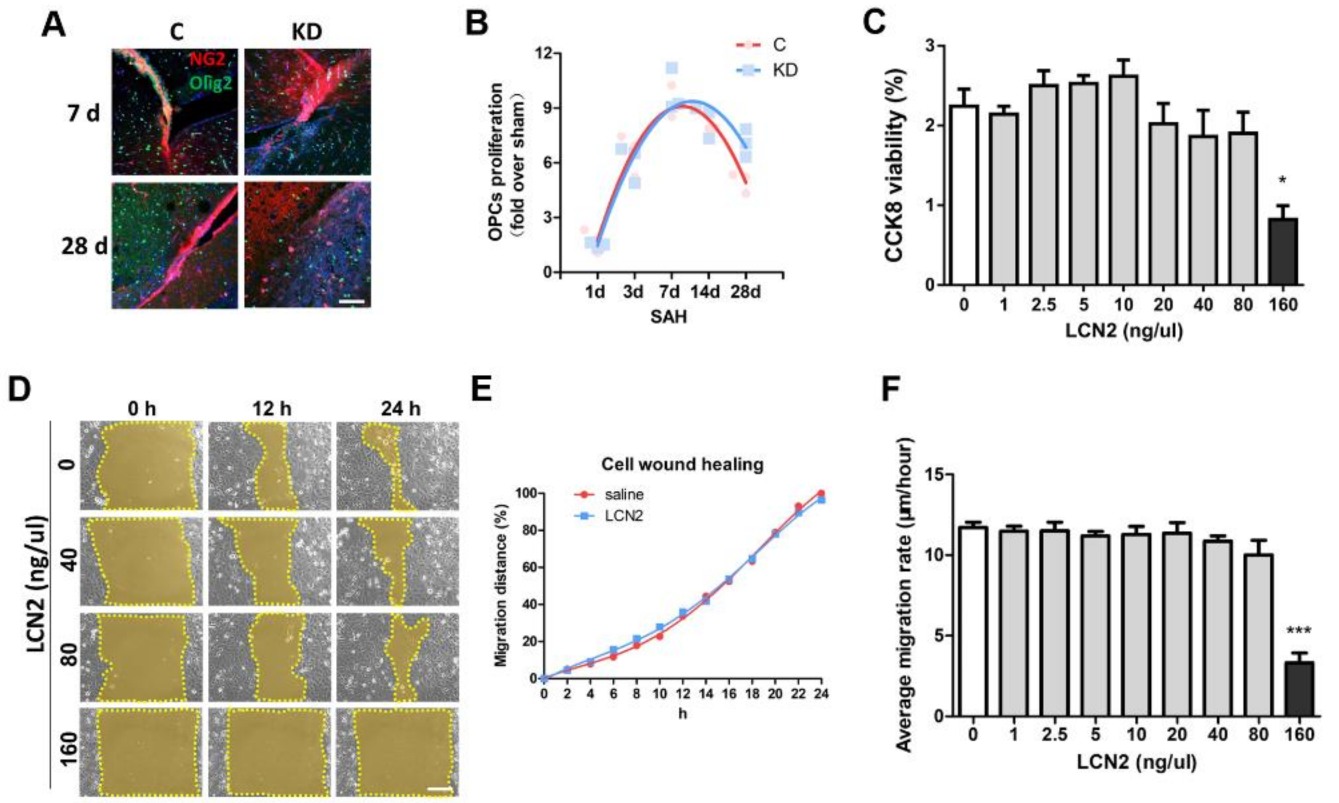

Fig. S5 Roles of LCN2 in the proliferation and migration of OPCs after SAH induction and LCN2 treatment.

A Images of OPCs (NG2$^+$Olig2$^+$) in the subventricular zone of control and LCN2^{KD} mice at 7 and 28 days after SAH. Scale bars, 25 μm. **B** OPC proliferation overlaid with polynomial best-fit regression curves (R^2 = 0.9401 and 0.9050) of control (pink) and LCN2^{KD} (light blue) mice at 1, 3, 7, 14, and 28 days after SAH. P = 0.8696; n.s. no significant difference, Kolmogorov–Smirnov test. **C** CCK-8 analysis of the viability of OPCs after treatment with 0, 1, 2, 5, 10, 20, 40, 80, or 160 ng/μL LCN2 (n = 3 per group). *P = 0.0389 *vs* 0 ng/μL LCN2 group, one-way ANOVA followed by Dunnett's multiple comparisons test. **D** Wound healing and live-cell imaging of OPC migration at 0, 12, and 24 h after treatment with 0, 40, 80, or 160 ng/μL LCN2 (n = 3 samples per group). The canary yellow field indicates an empty area without cells. **E** Analysis of the migration distance (1 – (mean distance at the

observed time point/mean distance at 0 h) × 100%) overlaid with polynomial best-fit regression curves (R^2 = 0.9941 and 0.9936) of OPCs treated with saline (0 ng/μL LCN2, magenta) and 40 ng/μL LCN2 (green). *P* = 0.6441; n.s. no significant difference, Kolmogorov–Smirnov test. **F** Average migration rates (μm/h) of OPCs treated with 0, 1, 2, 5, 10, 20, 40, 80, or 160 ng/μL LCN2 (*n* = 3 per group). ****P* = 0.0054 *vs* 0 ng/μL LCN2 group, one-way ANOVA followed by Dunnett's multiple comparisons test.

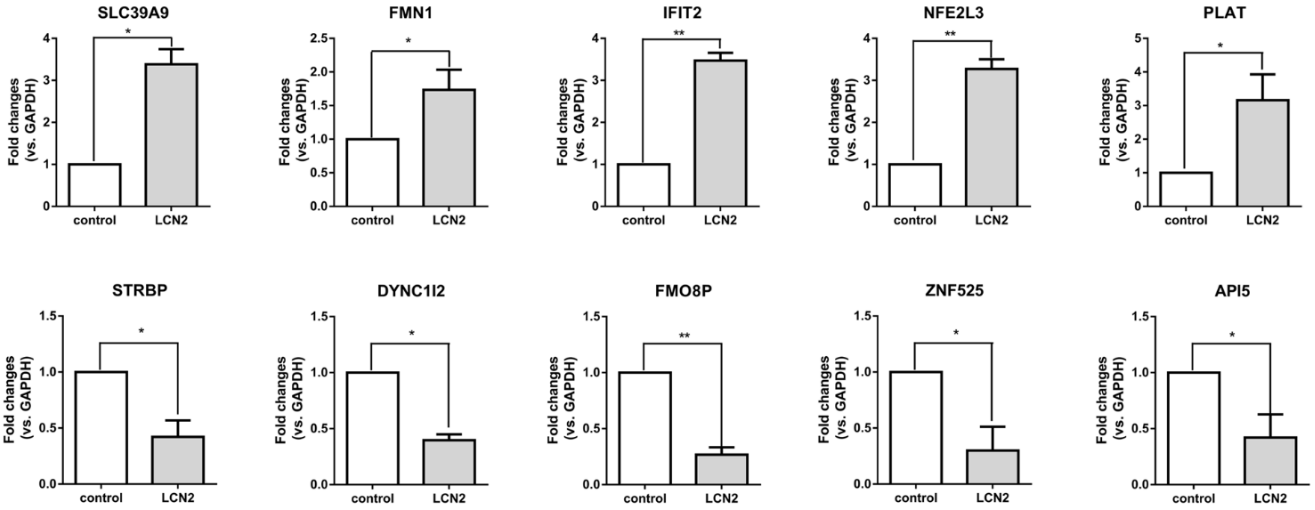

Fig. S6 qPCR analysis of the mRNA expression of some significantly differentially-expressed genes after LCN2 stimulation.

The five upregulated genes were SLC39A9, FMN1, IFIT2, NFE2L3, and PLAT, and the 5 downregulated genes were STRBP, DYNC1I2, FMO8P, ZFN525, and API5. *P* = 0.0111, 0.0284, 0.0029, 0.0051, 0.0446; 0.0253, 0.0438, 0.0464, 0.0429, and 0.0374, respectively, two-tailed Student's *t* test.

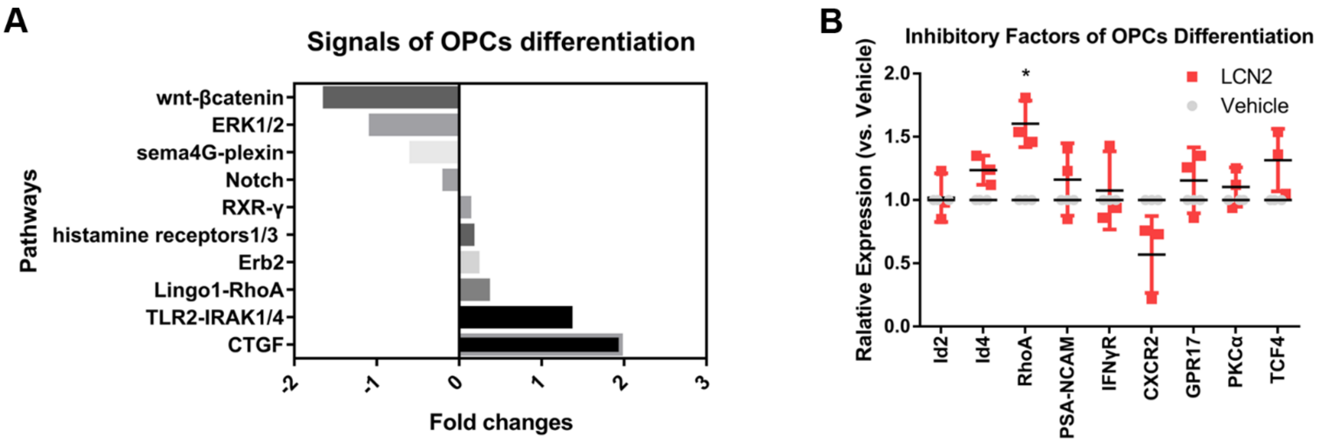

Fig. S7 Changes in signaling pathways and inhibitory factors regulating OPC differentiation after LCN2 stimulation.

A Several signaling pathways that regulate OPC differentiation are altered after LCN2 stimulation. Enriched genes are scored *versus* –Lg (p values). **B** qPCR analysis of several inhibitory factors regulating OPC differentiation after LCN2 stimulation. *P = 0.0146 *vs* vehicle, one-way ANOVA followed by Dunnett's multiple comparisons test.

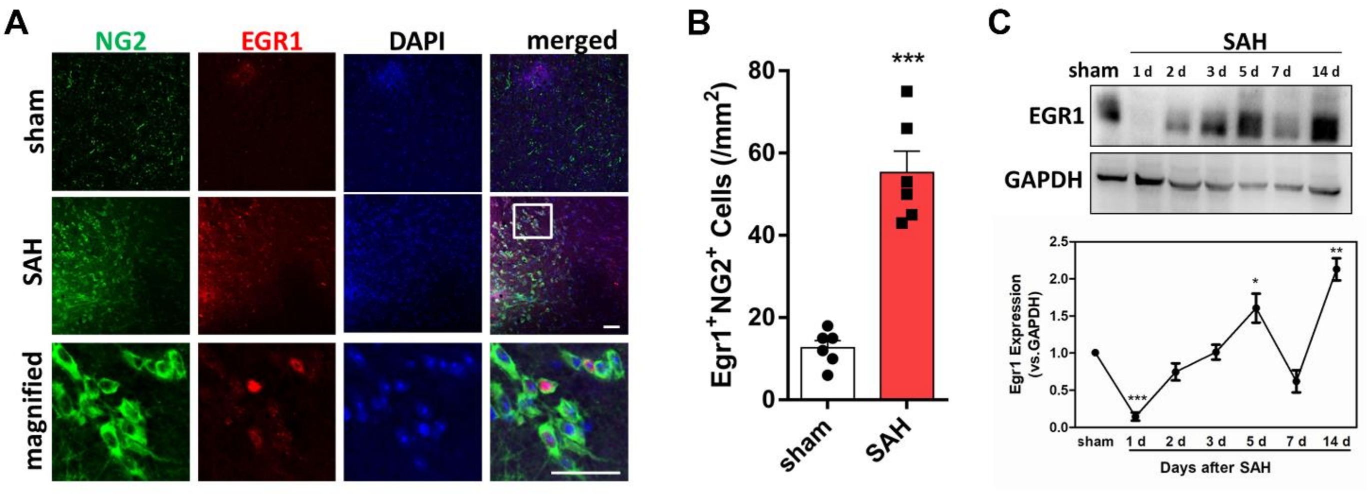

Fig. S8 Expression of EGR1 in OPCs after SAH.

A Co-localization of EGR1 and OPCs ($NG2^+$) at 5 days after SAH (scale bar, 50 μm). **B** Statistics of the number of $EGR1^+NG2^+$ cells per mm^2 (n = 6 fields per group). ***P = 0.0002, two-tailed Student’s t

test. **C** Western blots of mouse brain lysates at 1, 2, 3, 5, 7, and 14 days after sham or SAH surgery showing the levels of EGR1 (56 kDa), with GAPDH as a loading control. The figure below shows the expression of LCN2. *n* = 3 mice per group. **P* <0.05, ***P* <0.01, ****P* <0.001 *vs* sham group, one-way ANOVA followed by Dunnett's multiple comparisons test.

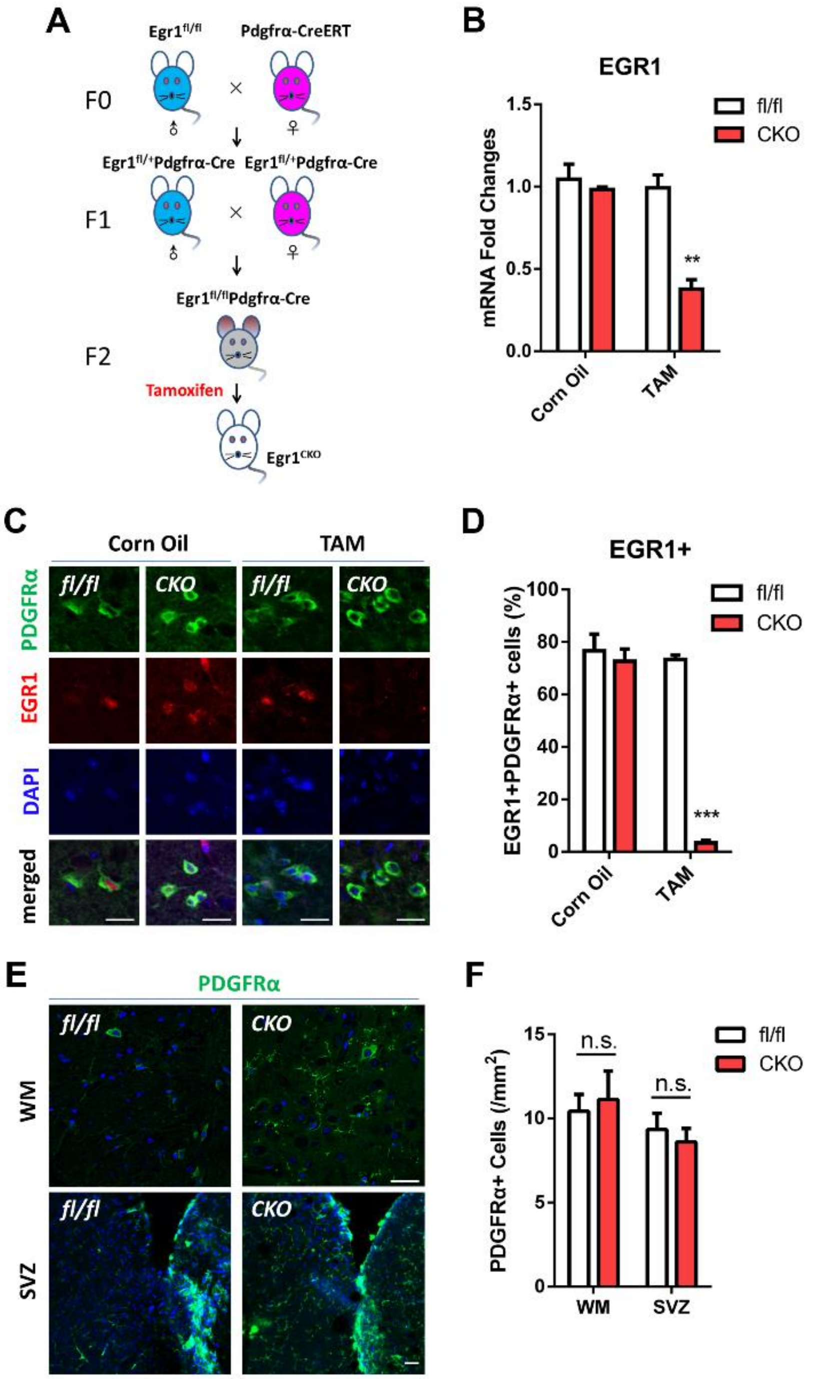

Fig. S9 Tamoxifen (TAM) successfully induces CKO of EGR1 in OPCs.

A Diagram of the process of CKO mouse production: (F0) male EGR1$^{fl/fl}$ mice were mated with female PDGFRa-CreErt mice, (F1) male EGR1$^{fl/+}$PDGFRa-Cre mice were mated with female syngeneic mice, and (F2) EGR1$^{fl/fl}$PDGFRa-Cre mice were administered tamoxifen (intraperitoneally) daily with continuing administration for 5 days beginning at P30 to induce a CKO mouse model. **B** qPCR analysis of the relative EGR1 mRNA expression in brain extracts from corn oil- (vehicle) and TAM-treated EGR1$^{fl/fl}$ and CKO mice (n = 3). **P = 0.0073, multiple t test. **C** Images showing the co-localization of EGR1 and OPCs (PDGFRa^{+}) in corn oil- and TAM-treated EGR1$^{fl/fl}$ and CKO mice at P60 (scale bars, 50 μm). **D** Proportions of EGR1^{+}PDGFRa^{+} cells among total PDGFRa^{+} cells (n = 6 fields per group). ***P = 0.0005, multiple t test. **E**. Images showing the co-localization of EGR1 and OPCs (PDGFRa^{+}) in EGR1$^{fl/fl}$ and CKO mice at P60 (scale bar, 75 μm). **F** Numbers of PDGFRa^{+} cells per square centimeter (n = 6 fields per group). P = 0.7401 in WM and 0.5853 in SVZ; n.s. no significant difference, multiple t test.

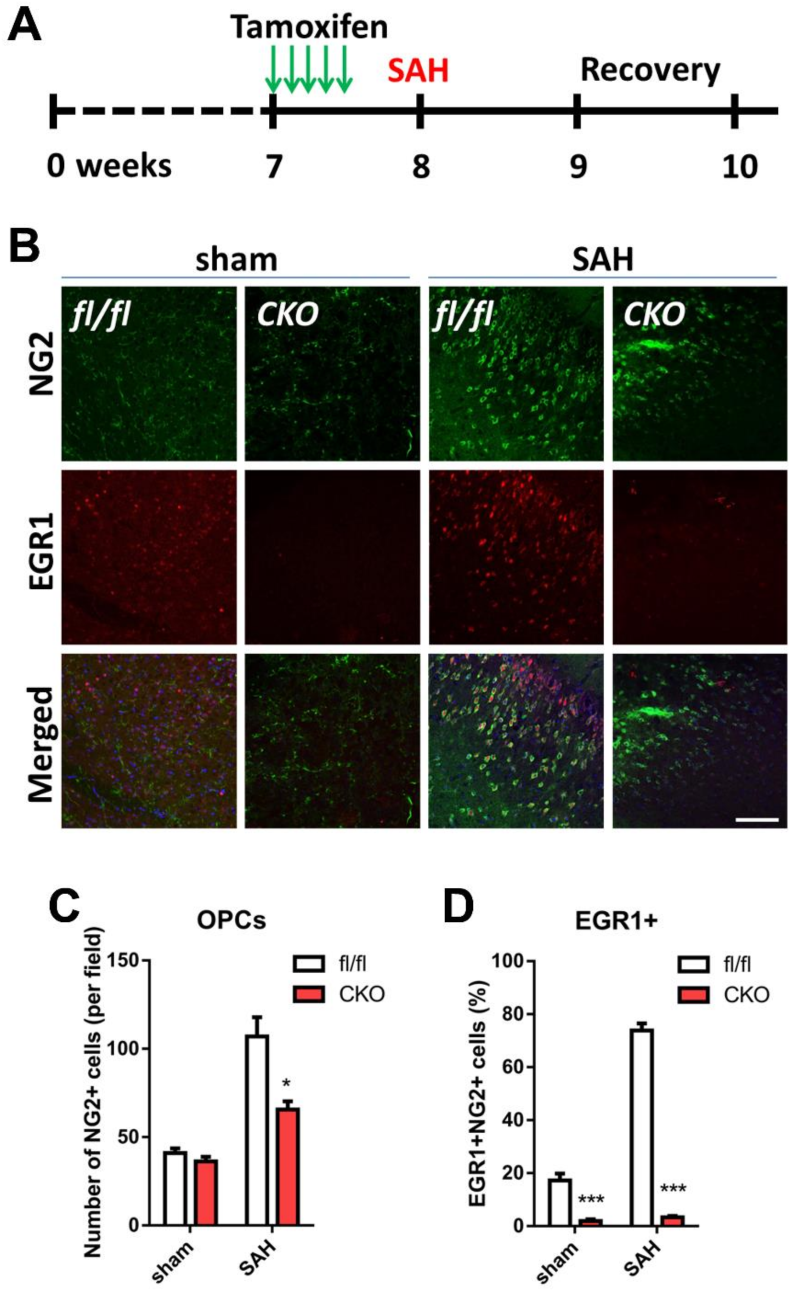

Fig. S10 CKO of EGR1 reduces the number of OPCs after SAH.

A Diagram of the timeline of TAM injection, SAH induction, and functional recovery after SAH. Each green arrow indicates one drug administration. **B** Images showing the co-localization of EGR1 and OPCs ($NG2^+$) in $EGR1^{fl/fl}$ and CKO mice at 7 days after sham or SAH surgery (scale bar, 100 μm). **C** Numbers of $NG2^+$ cells in each field of $EGR1^{fl/fl}$ and CKO mice at 7 days after sham or SAH surgery.

*P = 0.0238, multiple t test. **D** Proportions of $EGR1^{+}NG2^{+}$ cells among total $NG2^{+}$ cells (n = 6 fields per group) in $EGR1^{fl/fl}$ and CKO mice at 7 days after sham or SAH surgery. ***P = 0.0045 for the sham group and ***P = 0.0004 for the SAH group, multiple t test.

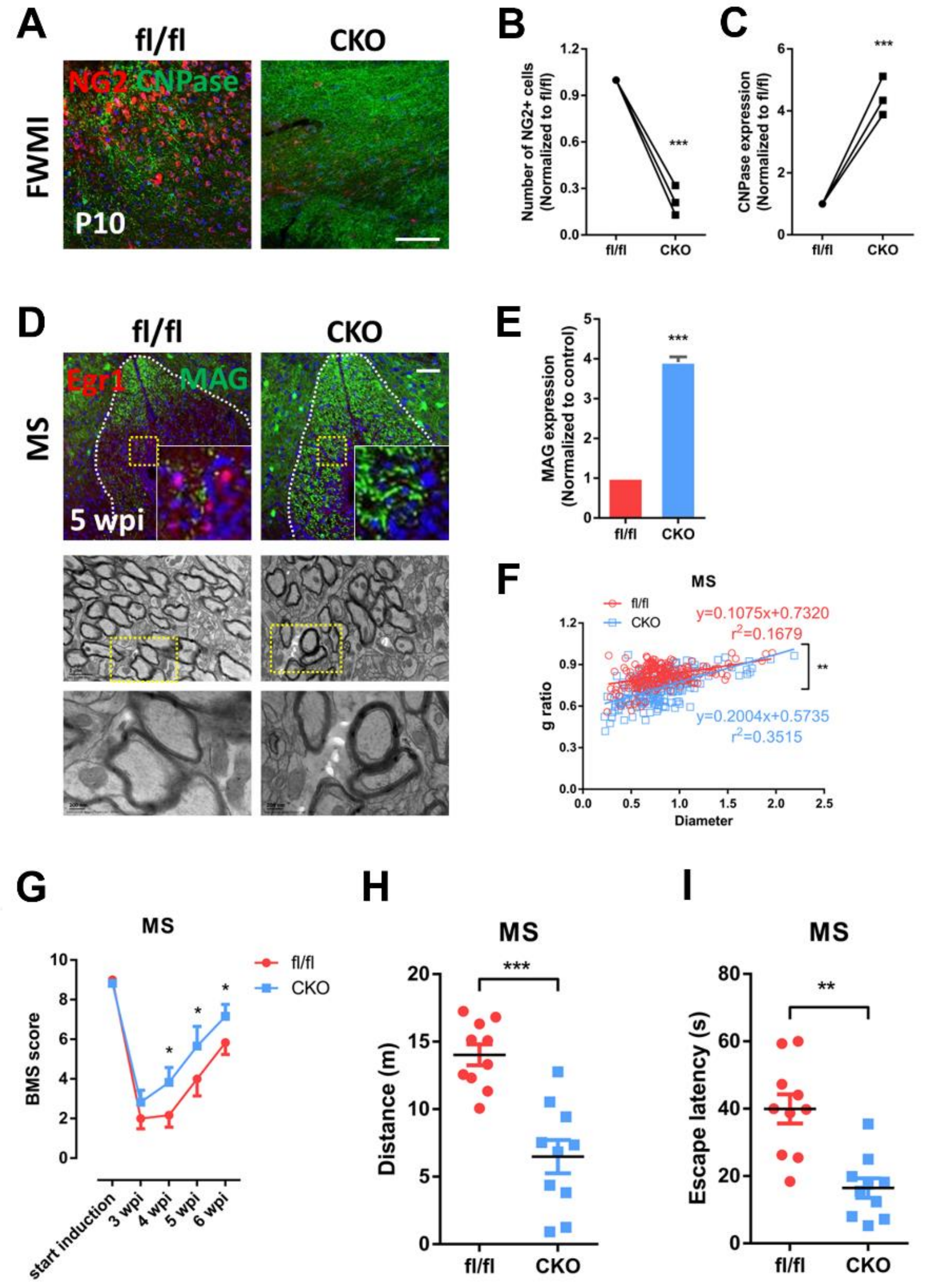

Fig. S11. CKO of EGR1 in OPCs promotes remyelination and functional recovery of MS and FWMI mice.

A Image showing NG2 (red) and CNPase (green) expression at 10 dpi (initiation of remyelination) in mice with FWMI (scale bar, 50 μm). **B** Normalized counts of $NG2^+$ cells in CKO mice compared with $EGR1^{fl/fl}$ mice (*n* = 4 per group) at 10 dpi of FWMI. ***P = 0.0001, two-tailed Student's *t* test. **C** Normalized CNPase expression in CKO mice compared with $EGR1^{fl/fl}$ mice (*n* = 4 per group) at 10 dpi of FWMI. ***P = 0.0002, two-tailed Student's *t* test. **D** Upper panel, images showing EGR1 (red) and MAG (green) expression in the spinal cord WM of $EGR1^{fl/fl}$ and CKO mice at 5 wpi of MS (scale bar, 75 μm). Lower panel, ultrastructure of the WM in $EGR1^{fl/fl}$ and CKO mice at 5 wpi of MS (scale bar, 2 μm). **E** Normalized MAG expression in fl/fl and CKO mice at 5 wpi of MS. ***P = 0.0002, two-tailed Student's *t* test; **F** G-ratio *versus* axon diameter in $EGR1^{fl/fl}$ and CKO mice at 5 wpi of MS. ***P = 0.0002, extra sum of squares F test between slopes. **G** BMS scores for $EGR1^{fl/fl}$ and CKO mice at 0 (induction), 3, 4, 5, and 6 wpi of MS. *P <0.05, two-way ANOVA followed by Sidak's multiple comparisons test. **H** Swimming distance before locating the platform of $EGR1^{fl/fl}$ and CKO mice with MS. ***P = 0.0001, two-tailed Student's *t* test. **I** Escape latency before finding the platform of $EGR1^{fl/fl}$ and CKO mice with MS. **P = 0.0042, two-tailed Student's *t* test.

Tables S1. Complete list of LCN2-regulated genes.
