# Supplementary material for: Lipocalin-2-Mediated Insufficient Oligodendrocyte Progenitor Cell Remyelination for White Matter Injury After Subarachnoid Hemorrhage via SCL22A17 Receptor/Early Growth Response Protein 1 Signaling
Source: Neurosci Bull. 2022 Jul 7;38(12):1457–75. doi: 10.1007/s12264-022-00906-w (PMC9723020; doi:10.1007/s12264-022-00906-w)
Supplement: Supplementary file 2 — Supplementary file2 (PDF 202 kb) [file 12264_2022_906_MOESM2_ESM.pdf]

Major Resources Tables

Animals (*in vivo* studies)

Species	Vendor or Source	Background Strain	Sex
mice	Third Military Medical University	C57BL/6	Male and female
mice	Jax library	PDGFR-α-creERT	female
mice	Model Animal Research Center of Nanjing University	$Egr1^{fl/fl}$	Male
mice	This paper	PDGFR-α-cre$Egr1^{fl/fl}$	Female and Male

Antibodies

Target antigen	species	Vendor or Source	Catalog #	Working concentration
NG2	Mouse monoclonal	Thermo Fisher Scientific	37-2700	1:100(IF)
	Rabbit polyclonal	Abcam	ab129051	1:100(IF)
LCN2	Goat Polyclonal	NOVUS	AF1757	1:100(IF)
	Rabbit polyclonal	Abcam	ab63929	1:100(IF); 1:1000(WB)
NF200	Rabbit polyclonal	Proteintech	18934-1-AP	1:100(IF)
	Mouse monoclonal	Thermo Fisher Scientific	13-1300	1:100(IF)
MBP	Mouse monoclonal	Proteintech	66003-1-Ig	1:200(IF)
	Rabbit polyclonal	Sigma-Aldrich	SAB2104172	1:200(IF)
MAG-mGFP	Conjugated	Sino biological	customized	2ug/100ul (IF)
CNPase	Mouse monoclonal	Abcam	ab6319	1:100(IF)
Olig-2	Rabbit polyclonal	NOVUS	NBP1-28667	1:100(IF)
CC-1	Mouse monoclonal	Merck Millipore	OP80	1:100(IF)
DM20	Mouse monoclonal	NOVUS	NBP1-50309	1:100(IF)
O4	Mouse monoclonal	NOVUS	NSC001	1:100(IF)
EGR1	Mouse monoclonal	Abcam	ab55160	1:100(IF)
	Rabbit polyclonal	CST	4154	1:100(IF)
	Goat polyclonal	R&D Systems	AF2818	1:1000(WB)
LRP2	Rabbit polyclonal	Proteintech	19700-1-AP	1:400(WB)
SLC22A17	Rabbit polyclonal	Thermo Fisher Scientific	PA5-20543	1:2000(WB)
DMBP	Rabbit polyclonal	US Biological	M9758-04	1:200(IF)
GFAP	Rabbit polyclonal	Proteintech	16825-1-AP	1:100(IF)
Iba1	Rabbit polyclonal	Proteintech	10904-1-AP	1:100(IF)
PDGFR-α	Rabbit monoclonal	Abcam	ab203491	1:100(IF)

Cultured Cells

Name	Vendor or Source	Sex (F, M, or unknown)
Oligodendrocyte precursor cell (OPCs)	Mouse primary	unknown
hOPCs	ScienCell	unknown

Primers

gene	sequence
Egr1	F: TGC TAA AGG GAA AGG GGA A
	R: TTG GGG AAG GGG AAG TG
Id2	F: CCC CAG AAC AAG AAG GTG
	R: GGC TGA CAA TAG TGG GAT G
Id4	F: TCA CTG CGC TCA ACA CC
	R: CCC CTC CCT CTC TAG TGC
RhoA	F: TGG AGC TGG GCT AAG TAA A
	R: CTC TGG GAG GGA ACC TG
PSA-NCAM	F: TCC GAG TCC CAG GTT TT
	R: CCC CGA AGT ACA GAA TGC
IFNγR	F: TGA CGG AAG TGA CGT AAG G
	R: AGG AGA AAG AGG AGA GCC A
CXCR2	F: CGC AAT GTG ACT TAA TGC C
	R: CTT TTG GGG CTC AGG TTT
GPR17	F: ATG CTG TTC GCC TCC TTC
	R: GGA CTT GTG GTC TCG GAT G
PKCα	F: CCA CAT CCA GGC AAG AAC
	R: GCA TCA GAA AGG GCA GAC
Tcf4	F: GCA GCA AGT CCG AGA AAG
	R: CTC CCA TTC CAG GGT GT
SLC39A9	F: TGC CTC ATC CCT CTC ATC
	R: GGC TGG ACC TTG AAC ATT T
FMN1	F: CGT GGG TTC TGG GCT AC
	R: GCT GTG GTC AGG ATG GAG
IFIT2	F: ATC TGC GGT ATG GCA ACT
	R: GGT GGA TGG CCT TGT CT
NFE2L3	F: GCA GAA CTG TCG TAA ACG C
	R: GGC CTA CCT TGG TCA TCT C
PLAT	F: CAG ACC GGG AAA AGG AG
	R: TGC CAT ATT AGC CCA TCA G
STRBP	F: ATG GAC TTG GAG CTG GTT T
	R: TAG CGT GGG CTC TTT TGT
DYNC1I2	F: TCG GGT GGT TAG TTG TTT G
	R: GAG GGG CAT CTT CAT TGT T
FMO8P	F: TCT ATC AAT GGA CAA TGG GA
	R: ATG GAA ATA ACA GCC TTC AAA
ZNF525	F: AAG GTT ATG GGG ACA ATG C

	R: CTA TGT TTC TGC TGT TTG CTG
API5	F: AAA TCG TAG TCG GGG AAG A
	R: CGG CAA TCC AAT GAG TAG TAG
TNFα	F: GGA AAG GAC ACC ATG AGC
	R: CCA CGA TCA GGA AGG AGA
IL-1β	F: TTG AGT CTG CCC AGT TCC
	R: TTT CTG CTT GAG AGG TGC T
CCL-2	F: TTT TCC CCT AGC TTT CCC
	R: GCA ATT TCC CCA AGT CTC T
IFNγ	F: AGC CCA GCA GCA TCT TC
	R: GAT TAC AGC CCA GAG AGC A
LRP2	F: TGC TTG GAT GTT CCC TGT
	R: AGG TTT AGG GGT CGG TTT T
SLC22A17	F: AGG TGG CAA GAA GAT CGG
	R: CAA AGC AGC AGG GAG GA
WNT2α	F: CAC CCA CCT TGA CGT TTC
	R: GGG CAG TTG ATT CTG CTT T
β-CATENIN	F: TGG GAC CTT GCA TAA CCT
	R: TTC ACC AGG GCA GGA AT
ERK1	F: GCT GGC TCA CCC CTA CC
	R: GGA AGC GTG CTG TCT CCT
ERK2	F: GGGGAGGTGGAGATGGT
	R: CGC CGA TGT ACT GCA ACT
TLR2	F: AAA CCG GAG AGA CTT TGC T
	R: TGT GTA TTC GTG TGC TGG A
IRAK4	F: AGG CAC TCA ACT TCA ACC A
	R: CCA CAT CCT GGC ACA CT
CTGF	F: GAA ATG CTG CGA GGA GTG
	R: CCC ACA GGT CTT GGA ACA
